# G protein-linked signaling pathways in bipolar and major depressive disorders

**DOI:** 10.3389/fgene.2013.00297

**Published:** 2013-12-23

**Authors:** Hiroaki Tomita, Mary E. Ziegler, Helen B. Kim, Simon J. Evans, Prabhakara V. Choudary, Jun Z. Li, Fan Meng, Manhong Dai, Richard M. Myers, Charles R. Neal, Terry P. Speed, Jack D. Barchas, Alan F. Schatzberg, Stanley J. Watson, Huda Akil, Edward G. Jones, William E. Bunney, Marquis P. Vawter

**Affiliations:** ^1^Department of Psychiatry and Human Behavior, University of CaliforniaIrvine, CA, USA; ^2^Department of Biological Psychiatry, Tohoku UniversitySendai, Japan; ^3^Functional Genomics Laboratory, Department of Psychiatry and Human Behavior, University of California IrvineCA, USA; ^4^Molecular and Behavioral Neurosciences Institute, University of MichiganAnn Arbor, MI, USA; ^5^Center for Neuroscience, University of CaliforniaDavis, CA, USA; ^6^HudsonAlpha Institute for BiotechnologyHuntsville, AL, USA; ^7^John A. Burns School of Medicine, University of HawaiiHonolulu, HI, USA; ^8^Department of Statistics, University of California BerkeleyCA, USA; ^9^Department of Psychiatry, Weill Cornell Medical CollegeNew York, NY, USA; ^10^Department of Psychiatry and Behavioral Sciences, Stanford UniversityPalo Alto, CA, USA

**Keywords:** G-protein coupled receptor (GPCR), transcriptome, bipolar disorder, major depressive disorder, GPR37, GPRC5B, cyclic AMP, phosphatidylinositol

## Abstract

The G-protein linked signaling system (GPLS) comprises a large number of G-proteins, G protein-coupled receptors (GPCRs), GPCR ligands, and downstream effector molecules. G-proteins interact with both GPCRs and downstream effectors such as cyclic adenosine monophosphate (cAMP), phosphatidylinositols, and ion channels. The GPLS is implicated in the pathophysiology and pharmacology of both major depressive disorder (MDD) and bipolar disorder (BPD). This study evaluated whether GPLS is altered at the transcript level. The gene expression in the dorsolateral prefrontal (DLPFC) and anterior cingulate (ACC) were compared from MDD, BPD, and control subjects using Affymetrix Gene Chips and real time quantitative PCR. High quality brain tissue was used in the study to control for confounding effects of agonal events, tissue pH, RNA integrity, gender, and age. GPLS signaling transcripts were altered especially in the ACC of BPD and MDD subjects. Transcript levels of molecules which repress cAMP activity were increased in BPD and decreased in MDD. Two orphan GPCRs, GPRC5B and GPR37, showed significantly decreased expression levels in MDD, and significantly increased expression levels in BPD. Our results suggest opposite changes in BPD and MDD in the GPLS, “activated” cAMP signaling activity in BPD and “blunted” cAMP signaling activity in MDD. GPRC5B and GPR37 both appear to have behavioral effects, and are also candidate genes for neurodegenerative disorders. In the context of the opposite changes observed in BPD and MDD, these GPCRs warrant further study of their brain effects.

## Introduction

The G-protein linked signaling system (GPLS) comprises G-proteins, G protein-coupled receptors (GPCRs), GPCR ligands, and downstream effector molecules. G-proteins interact with both GPCRs and downstream effectors such as cyclic adenosine monophosphate (cAMP), phosphatidylinositols, and ion channels. The GPLS is implicated in the pathophysiology and pharmacology of both major depressive disorder (MDD) and bipolar disorder (BPD). GPCRs are integral membrane proteins with key roles in numerous physiological cellular processes. GPCRs can signal ligand binding events by conformational changes that activate intracellular G proteins. Active G proteins in turn trigger intracellular downstream signaling pathways. Evidence suggests that altered GPCRs and their two major downstream signaling pathways, mediated by effectors such as cAMP and phosphatidylinositol, may be involved in the pathophysiology of BPD and MDD as well as in the mechanism of drug treatment for these disorders. Altered immunoreactivities and functional activities of proteins related to cAMP signaling pathways have been reported in the brain and peripheral blood cells of BPD patients (Jope et al., 1996; Karege et al., 1996; Gould and Manji, 2002; Chang et al., 2003). Also, mood stabilizers and antidepressants have been shown to affect cAMP and phosphatidylinositol signaling (Donati and Rasenick, 2003; Harwood, 2005; Xu et al., 2007; Racagni and Popoli, 2008). There are at least 210 GPCRs for which a natural ligand has been identified. There are 150 orphan GPCRs, with no known ligand or function (Wise et al., 2004), that might be relevant to downstream signaling dysregulation observed in BPD and MDD. Among the signal transmission systems associated with GPCRs, monoaminergic, and neuropeptidergic systems are believed to be dysregulated in BPD and MDD (Lieb et al., 2002; Elhwuegi, 2004; Carvajal et al., 2006; Ruhe et al., 2007).

Messenger RNA expression levels of a number of GPCRs, their ligands, and effector molecules involved in the GPLS pathways have been measured in post-mortem brains of BPD and MDD patients (Young et al., 1996; Spleiss et al., 1998; Caberlotto and Hurd, 2001; Kuromitsu et al., 2001; Zhou et al., 2001; Gurevich et al., 2002; Pandey et al., 2002; Agam et al., 2003; Chang et al., 2003; Dwivedi et al., 2004; Escriba et al., 2004; Lopez-Figueroa et al., 2004; Sherrin et al., 2004). Brain imaging studies have implicated anterior cingulate cortex (ACC) and the dorsolateral prefrontal cortex (DLPFC) in the pathophysiology of mood disorders (Harrison, 2002; Rogers et al., 2004; Frazier et al., 2005). In this study, the DLPFC and ACC were selected for microarray gene expression profiling of BPD and MDD patients focusing on the involvement of genes involved in GPLS pathways in these disorders and the effect of drug treatments. These cortical regions were selected because of the integral role of the ACC and DLPFC in the control of impulses, memory, learning, and hedonic evaluation, and depression. This study compares an a priori set of genes, in both mood disorders, using two cortical regions. There have been a number of previous studies comparing MDD and BPD by microarray analysis to controls, e.g., studies involving samples from the Stanley Foundation Neuropathology Brain collection (Kim and Webster, 2010). However, few studies have analyzed gene expression by microarray analysis of the Anterior Cingulate in both mood disorder groups, aside from studies involving the Pritzker Neuropsychiatric Disorders papers which involved selected pathways of glutamate, mitochondria, and growth factors (Evans et al., 2004; Choudary et al., 2005; Vawter et al., 2006). We hypothesized that there would be dysregulation of the GPLS in both mood disorders compared to controls, and that some overlap between the signature of each mood disorder would be found, given that most of our subjects were in a depressive state near the time of death.

## Materials and methods

### Subjects

The primary cohort consisted of 22 subjects, including seven healthy controls, six patients with bipolar disorder type I (BPD), and nine patients with MDD (Cohort A). The MDD results were validated with an additional independent cohort, including seven controls and five MDD patients (Cohort B). Toxicology data and/or recent clinical data were examined for the BPD and MDD subjects and detailed clinical information is summarized in Table 1. To minimize potential confounding effects of agonal events, we used three criteria in subject selection: brain tissue pH of >6.6, ribosomal RNA 28S/18S ratio of >1.5, and sudden death with the agonal period lasting less than 1 h. We have previously found large differences in gene expression values when using longer agonal periods, >1 h and as long as 1–2 days, and would have found large effects due only to mixing samples with long and short agonal duration (Li et al., 2004; Tomita et al., 2004; Vawter et al., 2006; Atz et al., 2007).

**Table 1:** **Demographics of two cohorts with mood disorder and matched controls, Cohorts A and B**.

**Gender (L)^a^**	**Age**	**Diagnosis**	**PMI**	**pH**	**28S/18S**	**SSRI^b^**	**Manner of death**	**Medications, time of death**
**COHORT A (BPD, MDD, CONTROL)**								
Male	9	BPD	11.25	6.91	1.55	NP	Exsanguination rupture of aorta	
Male (L)	3	BPD	9	7.12	2.1	NP	Hanging	Mood Stabilizer, Antipsychotic, Anticholinergic
Male (L)	26	BPD	19	6.92	1.81	NP	Carbon monoxide poisoning	Antipsychotic, Mood Stabilizer, Mood Stabilizer, Antianxiety Agent, Anticholinergic
Female	56	BPD	29	6.83	2.17	SSRI+	Acute strychnine intoxication	Antidepressant-Atyp^#^, Antidepressant-HCA^#^, Antianxiety Agent, Hypnotic, Antidepressant-SSRI
Male	52	BPD	28	7.05	2.36	NP	Acute hemorrhage slash wound to neck	
Male (L)	59	BPD	15.5	6.99	1.58	NP	Myocardial infarction	Mood Stabilizer
Female	72	MDD	21	7.13	2.28	NP	Pulmonary edema due to acute intoxication	Hypnotic, Antianxiety Agent, Antianxiety Agent, Antidepressant-HCA, Antidepressant-HCA
Male	19	MDD	18	7.11	2.17	NP	Asphyxiation due to hanging	
Male	58	MDD	24	6.93	2.26	SSRI+	Hanging	Antidepressant-SSRI, Antidepressant-Atyp
Male	49	MDD	31	7	1.96	SSRI+	Accidental overdose of propoxyphene, norpropoxyphene, amitriptyline, nortriptyline, sertaline, norsertaline and zolpidem	Antidepressant-SSRI, Antianxiety Agent, Hypnotic, Antidepressant-HCA
Male	46	MDD	27	6.91	2.07	NP	Occlusion of left anterior descending coronary artery	Hypnotic, Antianxiety Agent, Antianxiety Agent
Male	49	MDD	27	7.19	2.24	NP	Hanging	
Male	52	MDD	16	6.82	1.82	SSRI+	Acute myocardial infarction	Antidepressant-SSRI, Antidepressant-Atyp, Antipsychotic, Hypnotic
Female	48	MDD	37	6.95	1.86	SSRI+	Overdose morphine	Anticonvulsant, Antidepressant-SSRI, Antianxiety Agent
Male	39	MDD	27.5	6.79	1.77	SSRI+	Hanging	Anticonvulsant, Antidepressant-SSRI
Female	60	Control	24	6.99	1.77	NA	Myocardial infarction	
Male	70	Control	27	7.03	1.89	NA	Myocardial infarction	
Male	18	Control	22	6.97	2.53	NA	Freshwater drowning	
Male	58	Control	26	7.02	1.99	NA	Ventricular fibrillation	
Male	55	Control	18	6.89	2.2	NA	Myocardial infarction	
Male	45	Control	21	6.86	1.92	NA	Congestive heart failure	
Male	44	Control	23	6.87	1.9	NA	Myocardial infarction	
**COHORT B (MDD, CONTROL)**								
Male	35	MDD	24.75	7.04	2.14	NP	Hanging	Antidepressant-Atyp^#^
Male	47	MDD	29	7.25	2.08	NP	Hanging	
Female	80	MDD	15	6.68	2.21	NP	Hypertrophic cardiomyopathy	
Male	63	MDD	28.5	7.17	1.57	NP	Ruptured aortic abdominal aneurysm	
Male	66	MDD	32	7.05	2.07	SSRI+	Cardiac event	Antidepressant-SSRI
Male	77	Control	7.25	6.62	2.16	NA	Cardiac event	
Male	39	Control	18	6.81	1.67	NA	Cardiac arrhythmia	
Male	39	Control	30	7.02	1.88	NA	Electrocution	
Male	41	Control	22.5	7.01	2.08	NA	Severe coronary arteriosclerosis	
Male	65	Control	13.5	6.88	2.05	NA	Hemorrhagic pericarditis	
Male	49	Control	27.5	6.68	1.68	NA	Coronary Insufficiency	
Female	45	Control	32	6.6	1.71	NA	Bilateral extensive pulmonary embolism	

a L, prescribed lithium at time of death.

# Antidepressant-Atypical, Antidepressant-heterocyclic.

Mean + standard deviation of age: BPD, 47.5 ± 18.7; MDD, 48.0 ± 14.2; control, 50 ± 16.7.

Mean + standard deviation of PMI: BPD, 18.6 ± 8.4; MDD, 25.4 ± 6.5; control, 23.0 ± 3.1.

Mean + standard deviation of pH: BPD, 7.0 ± 0.1; MDD, 7.0 ± 0.1; control, 6.9 ± 0.1.

Mean + standard deviation of 28S/18S: BPD, 1.9 ± 0.3; MDD, 2.0 ± 0.2; control, 2.0 ± 0.3.

Mean ± standard deviation of age: MDD, 58.2 ± 17.5; control, 50.7 ± 14.7.

Mean ± standard deviation of PMI: MDD, 25.9 ± 6.6; control, 21.5 ± 9.1.

Mean ± standard deviation of pH: MDD, 7.0 ± 0.2; control, 6.8 ± 0.2.

Mean ± standard deviation of 28S/18S: MDD, 2.0 ± 0.3; control, 1.9 ± 0.2.

b NP, not prescribed SSRI; NA, not applicable for SSRI.

Brain tissue and clinical information were obtained by the UCI Brain Bank staff with the informed consent of decedents' next-of-kin, and were processed through a standard protocol approved by the local UCI Institutional Review Board (Tomita et al., 2004). None of the subjects included in this study had specific agonal conditions including cancer, hypoxia, coma, pyrexia, seizure, dehydration, hypoglycemia, multi-organ failure, skull fracture, ingestion of neurotoxic substances or prolonged agonal duration, which are known to affect tissue pH, RNA integrity and gene expression profiles in post-mortem brain (Li et al., 2004; Tomita et al., 2004). The variables for gender, age, post-mortem interval (PMI), tissue pH, and 28S/18S ratio were balanced between diagnostic and control groups. Patients who were prescribed lithium and selective serotonin reuptake inhibitor (SSRI) at the time of death are also indicated (Table 1).

### Microarray gene expression

The experimental procedures are described elsewhere (Evans et al., 2004; Tomita et al., 2004). Total RNA was extracted from ACC and DLPFC of Cohort A (Table 1), and purified with silica-based mini-spin columns (Qiagen, Valencia, California). The oligonucleotide microarray experiments were conducted utilizing Affymetrix U133A Gene Chips following the manufacturer's protocol (Affymetrix, Santa Clara, CA). To detect gene expression differences between diagnostic groups reliably, we replicated the experiments as follows: Cohort A used 22 subjects' total RNA from ACC which were processed at two laboratories (University of California, Irvine and University of California, Davis) and DLPFC that were processed independently (University of California, Davis and University of Michigan, Ann Arbor). For Cohort B 12 total RNA samples from ACC and DLPFC (additional 12 subjects) were processed at the same independent laboratories as cohort A. In summary, for each BPD subject there were duplicate arrays run, and for MDD subjects there were duplicate arrays run, plus an additional cohort of MDD subjects were run also with duplicate arrays.

### Data analyses

Since a considerable number of probe sequences in the Affymetrix original chip definition file (CDF) were found to incorrectly BLAST to other transcripts, a refined CDF based on an UniGene cluster was developed (Evans et al., 2004; Dai et al., 2005), and was used for data analyses presented in this paper. The refined CDF file is available on http://brainarray.mhri.med.umich.edu/brainarray/.

The focus of this study was 445 GPLS genes found on the Affymetrix arrays used in this study (Table S1). Signal intensities were extracted with Robust Multi-array Average (RMA) for each probe set and each subject. Gene-wise Pearson's correlation coefficients between experimental duplicates were calculated. Only the genes that were significantly correlated (*p* < 0.05) between experimental duplicates were considered to be reliably measured genes, and were subjected to downstream analyses. These genes were analyzed in a mixed-model ANOVA utilizing Partek Pro 6.0 (Partek, MO) for the main effect of the diagnostic classification (BPD, MDD, control) and for sex (Evans et al., 2004). We calculated the false discovery rate (FDR) in the microarray study by Benjamini-Hochberg step-down procedure using Partek Genomics setting the FDR to 0.05. Since the three diagnostic groups were well matched on age and pH (Table 1), the gene expression values were not tested by ANCOVA. However, in *post-hoc* analysis, we determined whether the tissue pH was correlated with any differentially expressed GPLS genes. The *post-hoc* analysis showed that there were no significant correlations found with tissue pH and differentially expressed GPLS genes. A *post-hoc* group comparison of subjects with SSRI treatment and without treatment was conducted in MDD subjects, and similar analysis for lithium was conducted for BPD subjects.

The hypergeometric distribution was used for calculating probabilities that the observed number of genes in each gene category for cAMP or PI signaling pathways (listed in Table S2) were detected as differentially expressed genes in the comparisons between patients and control. False discovery rate were evaluated by QVALUE (Storey and Tibshirani, 2003). Calculations for hypergeometric probabilities were performed using SISA online statistics package (http://www.quantitativeskills.com/sisa/distributions/hypergeo.htm).

### Quantitative PCR

For further technical evaluation of the microarray data, we evaluated mRNA expression levels by quantitative real-time reverse transcriptase PCR (qPCR) for the seven genes listed in Table 3. We followed up on results primarily that met FDR in cohort A for BPD, and both Cohorts A and B for MDD, except for RGS20, which we followed up based upon Cohort A only. These genes selected for qPCR analyses met the criteria of FDR at the level of accepting 5% false positives and percentage change greater than 20% in the ACC microarray results. The threshold cycle (C_*t*_) values for the target transcripts were normalized to averaged C_*t*_ values for two reference transcripts, Jagged 1 (JAG1) and solute carrier family 9 isoform 1 (SLC9A1), which showed equivalent expression levels among the three diagnostic groups (BPD, MDD, and control) throughout the 3 brain regions on our normalized microarray data. After DNase digestion and purification, total RNA (1 μg) samples from each of the 34 subjects in Cohorts A and B were used as a template for first-strand cDNA synthesis using poly-dT primer. The mRNA for each transcript was measured using the SybrGreen system with Prism model 7000-sequence detection instrument (Applied Biosystems, Foster City, California) and primer sequences listed in Table S1. The C_*t*_, which correlatedinversely with the target mRNA concentration, was measured as the cycle number at which the SybrGreen fluorescent signal increased abovea pre-set threshold level. The qPCR experiments were performed three times for each transcript, and the C_*t*_ triplicate values were averaged.

### *In situ* hybridization

The gene expression fold changes for GPRC5B and GPR37 showed an increase in BPD and decrease in MDD. We selected those genes for *in situ* hybridization (ISH) using the ACC regions from six representative subjects to evaluate the cellular localization of GPRC5B and GPR37 mRNA expression. A formal statistical analysis of the ISH data was not applied due to low subject number. ISH was performed following a protocol as described elsewhere (Neal et al., 2001; Lopez-Figueroa et al., 2004). Briefly, frozen ACC tissue blocks were cut into 12-μm sections on a cryostat. After fixation with 4% paraformaldehyde for 1 h, sections were hybridized overnight with a ^35^S-labeled antisense cRNA probes for GPRC5B and GPR37. Following hybridization, wash, RNase A treatment, dehydration and air-dry, sections were exposed to Kodak XAR-5 X-ray film for 21 days. Technical control studies were performed by (1) using ^35^S-labeled sense RNA probes for GPRC5B and GPR37, and (2) pre-treating tissue with RNase A before hybridizing with the antisense probes.

## Results

Significantly dysregulated GPLS genes that passed FDR (*p* < 0.05) in either Cohort A or B and either mood disorder, BPD or MDD, are listed in Table 2. For completeness, the uncorrected p-values are also shown, as some genes only met FDR for a single comparison. In general, equal numbers of GPLS genes passed FDR in BPD and MDD in the ACC (10 genes for BPD and 10 genes for MDD that passed FDR), compared with DLPFC (1 gene for BPD and 5 genes for MDD). Thus, about three fold more gene expression differences in mood disorder were found in the ACC showed compared to the DLPFC. The genes in Table 2 also showed non-significant correlations between gene expression and tissue pH (all *p*-values > 0.1) suggesting that residual pH-agonal effects on the expression levels of GPCR genes did not remain in the data set, even though complete elimination of these factors may not be possible with matching of subject's age and pH, and inclusion of subjects that had rapid deaths.

**Table 2:** **GPLS genes significantly dysregulated in anterior cingulate cortex by microarray**.

**Category**	**Anterior Cingulate Cortex**									
	**Symbol**	**BPD Cohort A**			**MDD Cohort A**			**MDD Cohort B**		
		**% change**	**Unadjusted *p*-value**	**FDR**	**% change**	**Unadjusted *p*-value**	**FDR**	**% change**	**Unadjusted *p*-value**	**FDR**
A. Cyclic AMP signaling pathway	NPY1R	24.4	0.000889	*p* < 0.05	21.9	0.000855	*p* < 0.05	−17.7	0.02108	
	NPY	33.0	1.48E−06	*p* < 0.05	3.6	0.37116		3.6	0.54313	
	SST	22.1	5.4E−05	*p* < 0.05	−13.3	0.002288	*p* < 0.05	−6.5	0.10886	
	GRM3	34.4	0.000113	*p* < 0.05	−10.9	0.081528		−17.9	0.01528	
	EDG1	−4.1	0.194276		−21.2	5.44E−07	*p* < 0.05	−10.4	0.00180	
	EDG2	18.2	0.000195	*p* < 0.05	4.8	0.1806		−4.5	0.22364	
	GNAI1	40.4	0.000289	*p* < 0.05	−2.4	0.740184		−9.0	0.26898	
	RGS20	15.6	0.005655	*p* < 0.05	−29.2	5.85E−06	*p* < 0.05	−13.9	0.00992	
	PDE1A	24.1	0.000599	*p* < 0.05	14.4	0.01141	*p* < 0.05	−8.0	0.19030	
	PDE8A	12.5	0.004505	*p* < 0.05	−23.1	3.96E−06	*p* < 0.05	−17.8	0.00138	
	PKIA	16.6	0.001318	*p* < 0.05	9.4	0.025332		−1.2	0.82836	
	PPP1CA	16.8	3.61E−06	*p* < 0.05	1.1	0.627546		1.1	0.79955	
	PPP1R3C	−15.4	0.000544	*p* < 0.05	−63.2	6.98E−13	*p* < 0.05	−21.4	0.00006	*p* < 0.05
B. Phosphatidylinositol signaling pathway	NTSR2	−1.4	0.741755		−18.6	0.000195	*p* < 0.05	−24.7	0.00012	*p* < 0.05
	EDNRB	−1.0	0.765956		−31.2	1.9E−08	*p* < 0.05	−15.9	0.00070	*p* < 0.05
	EDG2	18.2	0.000195	*p* < 0.05	4.8	0.1806		−4.5	0.22364	
	INPP5F	16.2	0.002445	*p* < 0.05	21.8	6.25E−05	*p* < 0.05	8.0	0.30847	
	ITPKB	11.5	0.035537		−12.3	0.015197	*p* < 0.05	−34.0	0.00010	*p* < 0.05
	INPP1	24.9	9.4E−06	*p* < 0.05	−1.1	0.761718		−7.4	0.04372	
	PIK3C2A	22.3	0.020892	*p* < 0.05	−35.9	0.000393	*p* < 0.05	−13.1	0.15764	
	PIK3C2B	14.8	0.001516	*p* < 0.05	−11.1	0.005757	*p* < 0.05	−1.1	0.81543	
	ITPR1	5.5	0.123943		17.4	2.66E−05	*p* < 0.05	10.5	0.15830	
	PRKCB1	−0.5	0.886704		11.5	0.00341	*p* < 0.05	5.7	0.15413	
C. Orphan GPCRs	GPR37	38.3	9.46E−08	*p* < 0.05	−40.5	8.39E−09	*p* < 0.05	−27.0	0.00003	*p* < 0.05
	GPRC5B	24.6	6.13E−06	*P* < 0.05	−38.3	2.94E−09	*p* < 0.05	−30.3	0.00004	*p* < 0.05
	GPR56	7.2	0.161404		−15.9	0.00261	*p* < 0.05	−14.9	0.00298	
	GPR125	−8.1	0.088684		−11.5	0.011594	*p* < 0.05	−0.3	0.94843	

The GPLS expression percent change differences (% change) by cohort for genes that met FDR in Cohort A or B. Blank FDR cells indicated those genes did not pass FDR.

### Cyclic AMP signaling pathway

**Figure 1:**
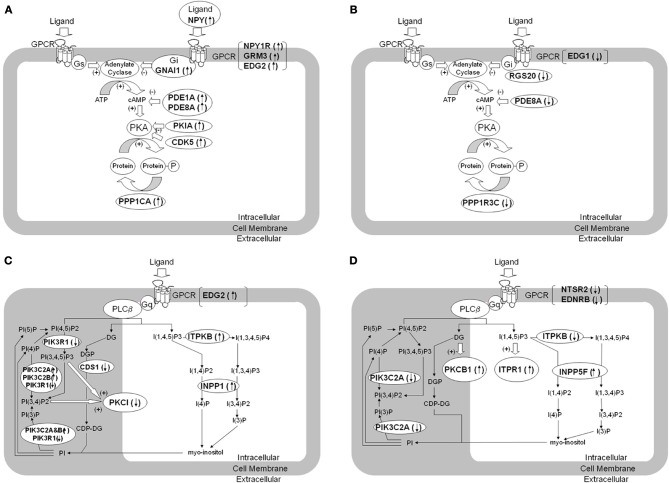
**Dysregulation of genes involved in cAMP- and phosphatidylinositol signaling pathways in brain tissue from patients with BPD and MDD.** The figure summarizes differentially expressed genes regulating signaling pathways using second messengers of cAMP **(A,B)** and phosphatidylinositol **(C,D)** in brain tissue from BPD **(A,C)** and MDD **(B,D)** compared to control subjects. The differentially expressed genes are denoted in italic and bold. **(A)** shows increased expression of transcripts related to suppression of cAMP mediated signaling in BPD. **(B)** shows decreased expression of transcripts related to suppression of cAMP mediated signaling in MDD. **(C)** shows altered expression of transcripts related to phosphatidylinositol mediated signaling in BPD. **(D)** shows altered expression of transcripts related to phosphatidylinositol mediated signaling in MDD. GNAI1, G protein alpha inhibiting activity 1; RGS20, Regulator of G-protein signaling 20; PDE1A, Phosphodiesterase 1A; PDE8A, Phosphodiesterase 8A; PKIA, Protein kinase A inhibitor alpha; PPP1CA, Protein phosphatase 1, catalytic alpha; PPP1R3C, Protein phosphatase 1, regulatory 3C; INPP5F, Inositol polyphosphate-5-phosphatase F; ITPKB, Inositol 1,4,5-trisphosphate 3-kinase B; INPP1, Inositol polyphosphate-1-phosphatase; CDS1, CDP-diacylglycerol synthase 1; PIK3C2A, Phosphoinositide-3-kinase catalytic 2A; PIK3C2B, Phosphoinositide-3-kinase catalytic 2B; PIK3R1, Phosphoinositide-3-kinase regulatory 1; PRKCI, Protein kinase C iota; ITPR1, Inositol 1,4,5-triphosphate receptor 1; PRKCB1, Protein kinase C beta 1; NPY, Neuropeptide Y; NPY1R, Neuropeptide Y receptor Y1; NTSR2, Neurotensin receptor 2; EDNRB, Endothelin receptor type B; GRM3, Metabotropic Glutamate receptor 3; EDG1, Endothelial differentiation GPCR 1; EDG2, Endothelial differentiation GPCR 2.

The genes involved in cAMP signaling pathways, which were differentially expressed in ACC of BPD patients compared to controls, are listed in Table 2. The cAMP signaling pathway is shown in Figure 1A. The data show that six significant transcripts out of 39 that repress cAMP signaling activity (Table 1) were increased in ACC from BPD patients at the transcriptional level. The group of molecules that act negatively on cAMP signaling activity was significantly over-represented in BPD based on a hypergeometric distribution (*p* = 0.037, *q* = 0.36). Thus, there was a nominal enrichment of genes that act negatively on cAMP signaling activity (G_*i*_ proteins, phosphodiesterase, protein kinase A inhibitors, and protein phosphatase) in BPD in the ACC.

In contrast, of those genes that repressed cAMP signaling activity in MDD (Figure 1B and Table 2) there was only one gene (PDE8A) differentially expressed, hence this signaling pathway was not over-represented (*p* > 0.05).

#### G_*s*_/G_*i*_-coupled GPCRs

Significant increases of NPY and NPY1R mRNA expression were found in BPD in ACC. GRM3 expression was also increased in ACC of BPD patients. G_*i*_/G_12_-linked EDG2 was increased in ACC of BPD, while G_*i*_-linked EDG1 was decreased in ACC of MDD. EDG1 and EDG2 regulate neuronal and glial functions, including neurite retraction, neurogenesis, and axonal guidance (Takuwa et al., 2002).

#### G proteins

Our data showed an increase of GNAI1 in the ACC of BPD, but no change in GNAI1 expression in the DLPFC of BPD. Consistent with our findings in DLPFC, immunoreactivity and functional activity of G_i_ protein is not altered in the frontal cortex of BPD (Young et al., 1993; Friedman and Wang, 1996). RGS20 was significantly decreased in ACC of MDD cases.

#### Adenylate cyclase and protein kinase A

Expression levels of transcripts coding adenylate cyclases and cAMP-dependent protein kinases (PKAs) were not altered in BPD and MDD compared with controls in our study, which is consistent with a previous study (Chang et al., 2003). The reported dysregulation in functional activities of adenylate cyclase and PKA (Gould and Manji, 2002) may not be due to alterations at the transcript level in the brain cortices.

#### Phosphatidylinositol signaling pathway

The phosphatidylinositol (PI) signaling pathway related genes which were differentially expressed in ACC of BPD patients are shown in Figure 1C and are summarized Table 2. The group of phosphatidylinositol 3-kinases was significantly over-represented in BPD based on a hypergeometric distribution (*p* = 0.042; *q* = 0.36).

The PI signaling pathway related genes which were differentially expressed in ACC of MDD patients compared with controls are summarized in Figure 1D and Table 2. Since antidepressants have been reported to affect PI signaling (Dwivedi et al., 2002; Quintero et al., 2005), the potential effect of drug treatment on the gene expression was considered. In cohort A, five MDD subjects were prescribed SSRI at the time of death, whereas remaining four MDD subjects were not prescribed and presumably not taking any SSRIs (Table 1). There were no significant differences in mRNA expressions of PKCB1, ITPR1 and INPP5F between SSRI-treated MDD and non-SSRI-treated MDD groups. However, ITPKB mRNA expression was significantly lower in the non-SSRI-treated MDD group than the SSRI-treated MDD group, which suggests that SSRIs may attenuate the altered expression in MDD toward levels observed in controls.

#### G_*q*_-coupled GPCRs

NTSR2 was decreased in ACC of BPD. NTSR2 mRNA and NTSR2 binding was reported to be down regulated in transgenic mice over expressing corticotrophin releasing factor, which is linked to anxiety, stress and depression (Peeters et al., 2004). EDNRB was also decreased in ACC of MDD. Endothelin is known to be a vasoconstrictor peptide as well as a neuromodulator regulating neuronal excitability. The data did not show any significant differences in expression levels of G_*q*_-linked monoamine GPCRs (serotonin-2 receptors and adrenergic alpha-1 receptors), which was consistent with previous microarray studies (Iwamoto et al., 2004; Konradi et al., 2004; Sibille et al., 2004; Aston et al., 2005).

#### Lithium and second messengers

The microarray data highlighted several target molecules of lithium treatment (Figure 1C). Lithium is known to block INPP1 (inositol polyphosphate-1-phosphatase) enzyme activity, and INPP1 mRNA expression was increased in ACC of BPD. Among the 6 BPD subjects, 3 BPD subjects treated with lithium at the time of death showed lower signal intensities for INPP1 and PIK3C2B compared with remaining non-lithium treated BPD subjects, although the difference did not reach significant changes. There is a modest genetic association between INPP1 and sensitivity to lithium treatment (Steen et al., 1998; Serretti, 2002). A second activity of lithium is activation of phosphatidylinositol 3-kinases and regulation of glycogen synthesis kinase 3 beta (GSK3B) activity (Sinha et al., 2005).

For MDD subjects, the mRNA expression of PKCB1, ITPR1, and INPP5F were increased in ACC, while ITPKB mRNA was decreased (Figure 1D). PKCB1 and ITPR1 are receptors for diacylglycerol (DG) and inositol 1, 4, 5-triphosphate (ITP), respectively, both of which are intracellular second messengers produced by PLC-beta through a G_*q*_ protein-dependent mechanism, while INPP5F and ITPKB metabolize ITP in a signal-terminating reaction. Previous reports suggest decreased PLC-beta activity in depressed patients (Pandey et al., 1997; Frey et al., 1998; Moore et al., 1999), and over expressed PKCB1 may down regulate PLC-beta activity (Pachter et al., 1992). The alterations in expression levels of the molecules interacting with DG and ITP may reflect functional impairments of G_*q*_ and PLC-beta in MDD.

### Orphan G protein-coupled receptors

The two GPCRs that showed the most consistent differential expression patterns through both mood disorder analyses were GPCR family C, group 5, member B (GPRC5B) and G protein-coupled receptor 37 (GPR37)as shown in Table 2 and validated by QPCR (Table 3). GPRC5B was significantly increased in ACC of BPD patients and in DLPFC of BPD. GPR37 was also significantly increased in ACC of BPD. GPRC5B was significantly decreased in ACC and DLPFC of MDD patients.

**Table 3:** **Quantitative PCR data summary of anterior cingulate cortex (ACC) genes in BPD or MDD that showed altered expression compared to the control group**.

**Symbol**	**Gene name**	**BPD vs. Control**				**MDD vs. Control**			
		**Microarray % change**	**Unadjusted *p*-value**	**qPCR % change**	**Unadjusted *p*-value**	**Microarray % change Cohort B**	**Unadjusted *p*-value**	**qPCR % change**	**Unadjusted *p*-value**
SST	Somatostatin	22.1	5.4 E−05^**^	10.1	*p* > 0.05	−6.5	0.108	NT	NT
GPRC5B	G protein-coupled receptor C−5−B	24.6	6.13E−06^**^	46.8	*p* < 0.05	−30.3	0.00004^**^	−54.0	*p* < 0.01
INPP1	Inositol polyphosphate-1-phosphatase	24.9	9.4E−06^**^	21.7	*p* < 0.05	−7.4	0.04372	NT	NT
NPY	Neuropeptide Y	33.0	1.48E−06^**^	37.6	*p* < 0.05	3.6	0.54313	NT	NT
GPR37	G protein-coupled receptor 37	38.3	9.46E−08^**^	54.1	*p* < 0.05	−27.0	0.00003^**^	−62.7	*p* < 0.05
RGS20	Regulator of G-protein signaling 20	15.6	0.005	NT	NT	−29.2^#^	5.85E−06^**^^#^	−36.9	*p* < 0.01
PPP1R3C	Protein phosphatase 1 regulatory subunit 3C	−15.4	0.0005	NT	NT	−21.4	0.00006^**^	−46.1	*p* < 0.01

These genes selected for qPCR analyses met the criteria of false discovery rate multiple testing correction at the level of accepting 5% false positives and percentage change greater than 20% in the anterior cingulate cortex microarray results in Cohort A for BPD, and in Cohort B for MDD. The qPCR analyses were run for seven representative GPLS selected from ligand peptides, GPCRs, and G protein regulators.

NT, not tested by qPCR as did not meet criteria of 20% change and passing FDR by microarray.

** Indicates a gene that passed FDR.

# RGS20 values shown are for Cohort A.

### Real time quantitative PCR

We followed up on results primarily that met FDR in cohort A for BPD, and both Cohorts A and B for MDD, except for RGS20, which we followed up based upon Cohort A meeting FDR while Cohort B met uncorrected p-value significance only. Nine GPLS transcripts were selected for validation, five for BPD and four for MDD. The qPCR results showed 100% concordance between qPCR and microarray fold change for BPD in ACC (Table 3) and 4/5 transcripts were statistically significant. Four GPLS related transcripts showed 100% concordance between qPCR and microarray fold change for MDD in ACC (Table 3) and 4/4 transcripts were statistically significant. NPY, GPRC5B, GPR37, and INPP1 were significantly increased in ACC of BPD, whereas GPRC5B, GPR37, RGS20, and PPP1R3C were significantly decreased in the ACC of MDD.

### *In situ* hybridization histochemistry

**Figure 2:**
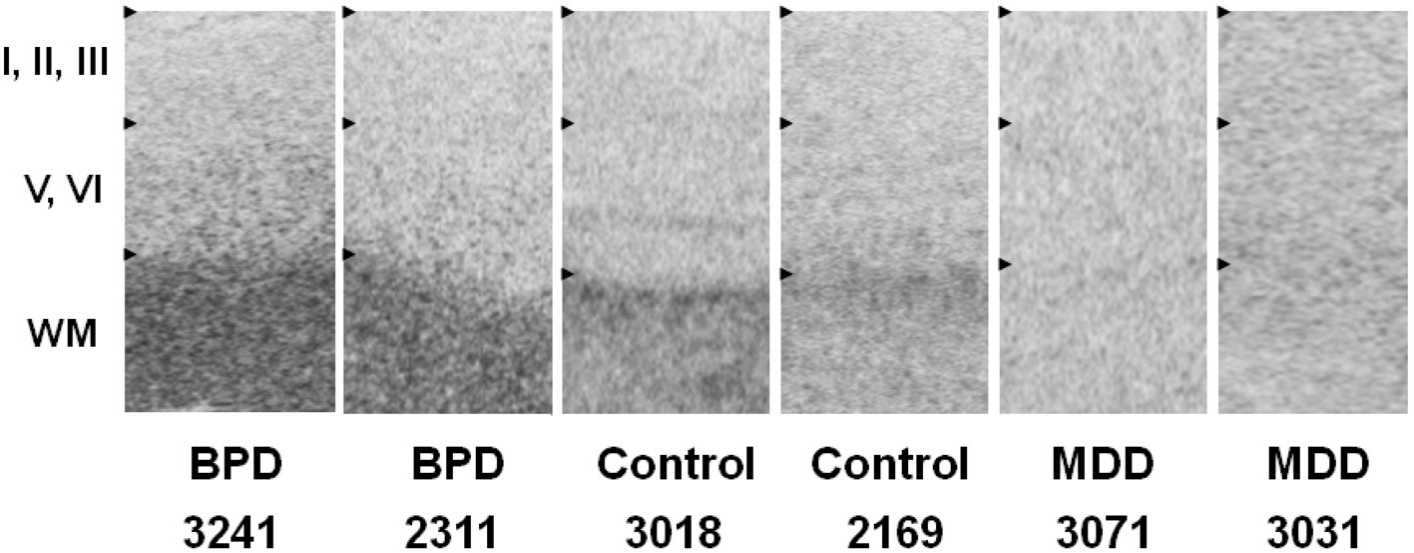
***In situ* hybridization images of GPR37 mRNA in representative BPD, MDD and control subjects.** GPR37 mRNA is preferentially expressed in subcortical white matter. Among six layers of cortical gray matter, GPR37 expression in deeper layers (V–VI) is relatively higher than in superficial layer (I–III). Expression levels in GPR37 is increased in the BPD subjects, and decreased in MDD subjects in subcortical white matter in anterior cingulate cortex tissue, compared to control subjects. WM, Subcortical white matter; BPD, Bipolar disorder; MDD, Major depressive disorder.

GPR37 mRNA was preferentially expressed in subcortical white matter. The sense probe hybridization signal showed background signal. GPR37 mRNA expression in subcortical white matter was higher in ACC of both of the BPD subjects compared to both of the control subjects, whereas GPR37 mRNA expression in subcortical white matter was lower in ACC of both of the MDD subjects compared to both of the control subjects (Figure 2). This preliminary data suggests that the significant differential microarray and QPCR expressions of GPR37 between BPD/MDD and controls may reflect altered expression levels of GPR37 mRNA in subcortical white matter. mRNA expression of GPRC5B was not specific in ACC samples tested, most likely due to strong background hybridization issues.

## Discussion

The results of the study of GPLS transcriptional profiling showed differences in BPD and MDD in both the cAMP- and phosphatidylinositol signaling pathways. The following pattern of alterations in GPLS were observed:

1. Transcriptional levels of molecules acting as negative regulators in cAMP signaling pathways are increased in BPD and decreased in MDD, which may compensate for the activated cAMP signaling activity in BPD and the blunted cAMP signaling activity in MDD,
2. Expression of PRKCB1, ITPR1, INPP5F, and ITPKB were altered in MDD, which may reflect functional impairments of G_*q*_ and PLC-beta in the disorder,
3. Expression of INPP1 and phosphatidylinositol 3-kinases were altered in BPD suggesting that these molecules may be involved in both the pathophysiology and the mechanism(s) of action of lithium treatment of BPD, and
4. Two orphan GPCR genes, GPRC5B and GPR37, showed significant increases in ACC of BPD, and decreases in ACC and DLPFC of MDD.

### Orphan G protein-coupled receptors

Among all GPCRs, the most consistent differential expression patterns were observed for GPRC5B and GPR37. GPRC5B was significantly increased in ACC and DLPFC of BPD, while significantly decreased in ACC and DLPFC of MDD patients. GPR37 was significantly increased in ACC of BPD, and significantly decreased in ACC and DLPFC of MDD patients. The consistent cortical alterations of GPRC5B and GPR37 expression observed in MDD patients suggest a role of these genes in the pathophysiology of MDD. So far, these two orphan GPCRs have not been highlighted as candidate susceptible genes for BPD and MDD, however previous microarray data supports decrease of both GPRC5B and GPR37 expressions in MDD patients (Aston et al., 2005). Our microarray data strongly suggests these genes might be involved in the pathophysiology of the disorders. GPR37 and GPRC5B are both implicated in Parkinson's disease (Marazziti et al., 2009, 2011), which has depression as one premorbid indicator, possibly concomitant with brain neurodegenerative processes.

GPR37 appears to facilitate dopamine neurotransmission, and reductions in brain GPR37 leads to behavioral alterations in conditioned place preferences to reinforcements such as cocaine or amphetamine (Marazziti et al., 2011). GPR37 (Class A receptor) contains a large extracellular domain which led to speculations that the ligand of this receptor may be a protein rather than a peptide (Leng et al., 1999). GPR37 is predominantly expressed in the brain (Zeng et al., 1997; Marazziti et al., 1998; Leng et al., 1999), especially in glial cells of the fiber tracts, Purkinje cells in cerebellum, neuronal cells in hippocampus CA3 region and substantianigra (Zeng et al., 1997; Imai et al., 2001). GPR37 has been shown to be involved in the dopaminergic system (Imai et al., 2001, 2002; Marazziti et al., 2004). GPR37 is also called Pael receptor (PAELR). Unfolded PAELR is a substrate of E3 ubiquitin ligase Parkin, and accumulation of Pael-R in the endoplasmic reticulum (ER) of dopaminergic neurons is considered to induce ER stress leading to neurodegeneration in Parkinson disease (Imai et al., 2002). Gpr37^−/−^ mice shows reduced striatal dopamine content and enhanced amphetamine sensitivity (Marazziti et al., 2004). Our ISH data suggest that the predominant expression of GPR37 mRNA in ACC occurred mainly in subcortical white matter. GPR37 is a substrate of parkin (PARK2), and its insoluble aggregates accumulate in brain tissue samples of Parkinson's disease patients, AKA PaelR (Marazziti et al., 2009).

GPRC5B is phylogenetically classified into a type C GPCR, however, in contrast to other type C genes, GPRC5B has a short N terminal domain (Brauner-Osborne and Krogsgaard-Larsen, 2000). QPCR based expression analysis shows GPRC5B is predominantly expressed in brain, with most abundant expression in neuronal cells of caudate, putamen, substantia nigra, thalamus and hippocampus and glial cells of the fiber tracts (Brauner-Osborne and Krogsgaard-Larsen, 2000; Robbins et al., 2002). Behavioral abnormalities exist in GPRC5B knock-outs, decreased spontaneous locomotor activity, and decreased responses to a new environment (Sano et al., 2011).

The expression patterns of GPRC5B and GPR37 suggest that both of these orphan GPCRs may be involved in various cellular functional activities in the brain including neuronal and glial cells. Functional characterization of GPRC5B and GPR37 may provide new insights into the pathophysiology of mood disorder, and potential novel strategies for developing methods for diagnoses and treatment of the disorders.

There are limitations to this post-mortem study of GPLS pathway expression. We conducted seven qPCR validations of genes that passed FDR, and although a small number, those genes did represent large enough percent fold changes to validate by qPCR. Other technologies could be used that are less costly, such as Nanostring or Fluidigm for additional validation studies, which are now being conducted on larger sample sizes. In addition, we do not have confirmed toxicological analysis of all drugs of abuse and therapeutic levels to rule out these effects in explaining case—control expression differences. Finally, we are aware that small residual agonal effects might exist that are not corrected in this analysis, however we did confirm that the present data presented in Table 2, was not due to the effects of age or pH on gene expression.

## Summary

This study highlights transcript profile differences in GPLS pathways in both BPD and MDD. Since sample sizes were low, we also used a second independent cohort in the case of MDD gene expression to check concordance. We relied upon duplicate microarray analysis at independent laboratories, and used gene expression values that were significantly correlated between sites, and employed FDR to select genes to advance for qPCR experimental validations.

The disturbances observed in cAMP- and phosphatidylinositol- pathway regulation may be due to differential activation of individual GPCRs and individual differences in ligand- receptor sensitivity, suggesting that functional and genetic characterization of these candidate genes may provide further understanding of the pathophysiology of mood disorders. Further studies will also be required to investigate additional GPCR signaling pathway features such as receptor phosphorylation, beta-arrestin recruitment, receptor desensitization, and internalization, and beta-gamma mediated signaling. The results of the present study suggest that future functional activity studies of the GPLS pathways would elucidate the relationship of the observed transcript changes to protein levels and functional outcomes in mood disorders.

### Conflict of interest statement

The authors declare that the research was conducted in the absence of any commercial or financial relationships that could be construed as a potential conflict of interest.
