# Supplementary material for: G protein-linked signaling pathways in bipolar and major depressive disorders
Source: Front Genet. 2013 Dec 23;4:297. doi: 10.3389/fgene.2013.00297 (PMC3870297; doi:10.3389/fgene.2013.00297)
Supplement: Supplementary file 1 [file DataSheet1.DOCX]

Supplementary Table S1. Primer sequences for qPCR results shown in Table 3.

| **Symbol** | **Gene Name** | **Forward primer** | **Reverse primer** |
| --- | --- | --- | --- |
| **Target genes** | |  |  |
| SST | Somatostatin | AGACTTTCACATCCTGTTAGCTTTCTTA | GACTTGGAGGATTAGGGAAGAGAGA |
| NPY | Neuropeptide Y | TGCAATGTGGTGATGGGAAA | GCATTGGTAGGATGGGTGGAT |
| GRM3 | Metabotropic glutamate receptor 3 | GGTCTGACATGGTCAGTCTACTAAAAAA | CCTAAATTAGTTACAACAAGGACCTACAAA |
| GPRC5B | G protein-coupled receptor C-5-B | AGGGCTGTTTTCATGGAACCT | TTCCTGGATGGAATGGATCAC |
| GPR37 | G protein-coupled receptor 37 | CAAACAAATAAATCTGACCCAACCA | TCTCGCCTTTCAGTACCATACG |
| RGS20 | Regulator of G-protein signalling 20 | CCACTTTTATTTTGCATGAGTTTGG | AACCGAGCTTGCTGGACAGA |
| PDE1A | Phosphoserine 1A | TCTTGCTTTGAACCCTGTCA | CTGTCATTCTGTTCCCATGC |
| PDE8A | Phosphoserine 8A | AATCGCTCACTTGGGAACAC | TTGAAGTTGGCTTTGGCTCT |
| PPP1R3C | Protein phosphatase 1 regulatory subunit 3C | TCCCAAAAGACTTGCCCATT | GATACCTTCTCATTTGCCATTGACT |
| INPP1 | Inositol polyphosphate-1-phosphatase | CTGAGCCTCCTGGTCCAAAA | CCGAAACAGTTTCACAGTTCAGTT |
| **Reference genes** | |  |  |
| JAG1 | Jagged 1 | CCTTGCTGAGCTCTGTCTTAACTGT | TTCCGCAGGCACCAGTAGA |
| SLC9A1 | Solute carrier family 9, isoform 1 | GGTCCTCGGCCCATCTG | TTGGTACGTGGTTGTCGATGTC |

Forward and reverse primers for the genes were designed by Primer Express version 1.5 (Applied Biosystems).

Gene titles: Somatostatin (SST), neuropeptide Y (NPY), metabotropic glutamate receptor 3 (GRM3), G protein-coupled receptor C-5-B (GPRC5B), G protein-coupled receptor 37 (GPR37), regulator of G-protein signaling 20 (RGS20), phosphodiesterase 1A (PDE1A) and phosphodiesterase 8A (PDE8A), protein phosphatase 1 regulatory subunit 3C(PPP1R3C) and inositol polyphosphate-1-phosphatase (INPP1).

Supplementary Table S2. Classification of genes involved in GPLS pathways that passed FDR. The GPLS system consists of genes for neuropeptide ligands for GPCRs, G proteins including G protein alpha subunits-s and -i (G_i_ and G_s_), regulators of G proteins, genes involved in cAMP signaling pathway (adenylate cyclase, phosphodiesterases, protein kinase A, modulators of protein kinase A, protein phosphatases), phosphatidylinositol signaling pathway (G protein alpha subunit-q (G_q_), phospholipase C beta (PLC-beta), phosphatidylinositol metabolism, protein kinase C and inositol triphosphate receptor), and ion-channels some of which are directly affected by G-protein binding.

| General category | Specific category | Gene name | Gene symbol | Alternative symbol | Dysregulated brain region |
| --- | --- | --- | --- | --- | --- |
| GPCR | GPCR-VIP and PACAP | Adenylate cyclase activating polypeptide 1 receptor type I | ADCYAP1R1 |  |  |
| GPCR | GPCR-adenosine | Adenosine A1 receptor | ADORA1 |  |  |
| GPCR | GPCR-adenosine | Adenosine A2a receptor | ADORA2A |  |  |
| GPCR | GPCR-adenosine | Adenosine A2b receptor | ADORA2B |  |  |
| GPCR | GPCR-adenosine | Adenosine A3 receptor | ADORA3 |  |  |
| GPCR | GPCR-adrenergic | Adrenergic alpha-1A receptor | ADRA1A |  |  |
| GPCR | GPCR-adrenergic | Adrenergic alpha-1B receptor | ADRA1B |  |  |
| GPCR | GPCR-adrenergic | Adrenergic alpha-1D receptor | ADRA1D |  |  |
| GPCR | GPCR-adrenergic | Adrenergic alpha-2A receptor | ADRA2A |  |  |
| GPCR | GPCR-adrenergic | Adrenergic alpha-2B receptor | ADRA2B |  |  |
| GPCR | GPCR-adrenergic | Adrenergic alpha-2C receptor | ADRA2C |  |  |
| GPCR | GPCR-adrenergic | Adrenergic beta-1 receptor | ADRB1 |  | DLPFC |
| GPCR | GPCR-adrenergic | Adrenergic beta-2 receptor | ADRB2 |  |  |
| GPCR | GPCR-adrenergic | Adrenergic beta-3 receptor | ADRB3 |  |  |
| GPCR | GPCR-angiotensin | Angiotensin II receptor type 1 | AGTR1 |  |  |
| GPCR | GPCR-angiotensin | Angiotensin II receptor type 2 | AGTR2 |  |  |
| GPCR | GPCR-Apelin | Angiotensin II receptor-like 1 / Apelin receptor | AGTRL1 | APJ /APLNR |  |
| GPCR | GPCR-AVP | Arginine vasopressin receptor 1A | AVPR1A |  |  |
| GPCR | GPCR-AVP | Vasopressin V1b receptor | AVPR1B |  |  |
| GPCR | GPCR-AVP | Arginine vasopressin receptor 2 | AVPR2 |  |  |
| GPCR | GPCR-Class B Orphans | Brain-specific angiogenesis inhibitor 1 | BAI1 |  |  |
| GPCR | GPCR-Class B Orphans | Brain-specific angiogenesis inhibitor 2 | BAI2 |  |  |
| GPCR | GPCR-Class B Orphans | Brain-specific angiogenesis inhibitor 3 | BAI3 |  |  |
| GPCR | GPCR-Bradykinin | Bradykinin receptor B1 | BDKRB1 |  |  |
| GPCR | GPCR-Bradykinin | Bradykinin receptor B2 | BDKRB2 |  |  |
| GPCR | GPCR-bombesin | Bombesin receptor type 3 | BRS3 |  |  |
| GPCR | GPCR-ANAPHYLATOXIN | Complement component 3a receptor 1 / Anaphylaxin receptor C3AR | C3AR1 |  |  |
| GPCR | GPCR-ANAPHYLATOXIN | Complement component 5 receptor 1 / Anaphylaxin receptor C5AR | C5R1 |  |  |
| GPCR | GPCR-CALCITONIN | Calcitonin receptor | CALCR | CTR1 |  |
| GPCR | GPCR-Calcium sensing | Calcium-sensing receptor | CASR |  |  |
| GPCR | Non-signalling 7TM chemokine-binding proteins | Chemokine binding protein 2 / C-C chemokine receptor CCR10 | CCBP2 | CCRL1 |  |
| GPCR | GPCR-CCK | Cholecystokinin A receptor | CCKAR |  |  |
| GPCR | GPCR-CCK | Cholecystokinin B receptor | CCKBR |  |  |
| GPCR | GPCR-chemokine | Chemokine (C-C motif) receptor 1 | CCR1 |  |  |
| GPCR | GPCR-chemokine | Chemokine (C-C motif) receptor 3 | CCR3 |  |  |
| GPCR | GPCR-chemokine | Chemokine (C-C motif) receptor 4 | CCR4 |  |  |
| GPCR | GPCR-chemokine | Chemokine (C-C motif) receptor 6 | CCR6 |  |  |
| GPCR | GPCR-chemokine | Chemokine (C-C motif) receptor 7 | CCR7 |  |  |
| GPCR | GPCR-chemokine | Chemokine (C-C motif) receptor 8 | CCR8 |  |  |
| GPCR | GPCR-chemokine | Chemokine (C-C motif) receptor 9 | CCR9 |  |  |
| GPCR | GPCR-Class B Orphans | CD97 antigen | CD97 |  |  |
| GPCR | GPCR-Class B Orphans | Cadherin, EGF LAG seven-pass G-type receptor 1 | CELSR1 |  |  |
| GPCR | GPCR-Class B Orphans | Cadherin, EGF LAG seven-pass G-type receptor 2 | CELSR2 |  |  |
| GPCR | GPCR-acetylcholine (Muscarinic) | Muscarinic acetylcholine receptor M2 | CHRM2 |  |  |
| GPCR | GPCR-acetylcholine (Muscarinic) | Muscarinic acetylcholine receptor M3 | CHRM3 |  |  |
| GPCR | GPCR-acetylcholine (Muscarinic) | Muscarinic acetylcholine receptor M4 | CHRM4 |  |  |
| GPCR | GPCR-acetylcholine (Muscarinic) | Muscarinic acetylcholine receptor M5 | CHRM5 |  |  |
| GPCR | GPCR-Class A Orphans | Chemokine-like receptor 1 | CMKLR1 |  |  |
| GPCR | GPCR-Cannabinoid | Cannabinoid receptor 1 | CNR1 |  |  |
| GPCR | GPCR-Cannabinoid | Cannabinoid receptor 2 | CNR2 |  |  |
| GPCR | GPCR-CRHR | Corticotropin releasing hormone receptor 1 | CRHR1 |  |  |
| GPCR | GPCR-CRHR | Corticotropin releasing hormone receptor 2 | CRHR2 |  |  |
| GPCR | GPCR-chemokine | Chemokine (C-X3-C motif) receptor 1 | CX3CR1 |  |  |
| GPCR | GPCR-chemokine | Chemokine (C-X-C motif) receptor 3 | CXCR3 |  |  |
| GPCR | GPCR-chemokine | Chemokine (C-X-C motif) receptor 4 | CXCR4 |  |  |
| GPCR | GPCR-chemokine | Chemokine (C-X-C motif) receptor 5 / Burkitt lymphoma receptor 1 | CXCR5 | BLR1 |  |
| GPCR | GPCR-chemokine | Chemokine (C-X-C motif) receptor 6 | CXCR6 |  |  |
| GPCR | GPCR-Leukotriene | Cysteinyl leukotriene receptor 2 | CYSLTR2 |  |  |
| GPCR | GPCR-DRD | Dopamine receptor D1 | DRD1 |  |  |
| GPCR | GPCR-DRD | Dopamine receptor D2 | DRD2 |  |  |
| GPCR | GPCR-DRD | Dopamine receptor D3 | DRD3 |  |  |
| GPCR | GPCR-DRD | Dopamine receptor D4 | DRD4 |  |  |
| GPCR | GPCR-DRD | Dopamine receptor D5 | DRD5 |  |  |
| GPCR | GPCR-Class A Orphans | Epstein-Barr virus induced gene 2 / lymphocyte-specific G protein-coupled receptor | EBI2 |  |  |
| GPCR | GPCR-Lysophospholipid | Endothelial differentiation / Sphingolipid G-protein-coupled receptor, 1 | EDG1 | S1PR1 | ACC |
| GPCR | GPCR-Lysophospholipid | Endothelial differentiation G-protein-coupled receptor 2 / Lysophosphatidic acid receptor 1 | EDG2 | LPAR1 | ACC |
| GPCR | GPCR-Lysophospholipid | Endothelial differentiation G-protein-coupled receptor 4 / Lysophosphatidic acid receptor 2 | EDG4 | LPAR2 |  |
| GPCR | GPCR-endothelin | Endothelin receptor type A | EDNRA |  |  |
| GPCR | GPCR-endothelin | Endothelin receptor type B | EDNRB |  | ACC |
| GPCR | GPCR-Class B Orphans | EGF, latrophilin and seven transmembrane domain containing 1 | ELTD1 |  |  |
| GPCR | GPCR-Class B Orphans | Egf-like module containing, mucin-like, hormone receptor-like 1 | EMR1 |  |  |
| GPCR | GPCR-Class B Orphans | Egf-like module containing, mucin-like, hormone receptor-like 2 | EMR2 |  |  |
| GPCR | GPCR-Class B Orphans | Egf-like module containing, mucin-like, hormone receptor-like 3 | EMR3 |  |  |
| GPCR | GPCR-PAR | Coagulation factor II (thrombin) receptor / Protease-activated receptor 1 | F2R | PAR1 |  |
| GPCR | GPCR-PAR | Coagulation factor II (thrombin) receptor-like 1 / Protease-activated receptor 2 | F2RL1 | PAR2 |  |
| GPCR | GPCR-PAR | Coagulation factor II (thrombin) receptor-like 2 / Protease-activated receptor 3 | F2RL2 | PAR3 |  |
| GPCR | GPCR-PAR | Coagulation factor II (thrombin) receptor-like 3 / Protease-activated receptor 4 | F2RL3 | PAR4 |  |
| GPCR | GPCR-formyl peptide | Formyl peptide receptor 1 | FPR1 | FPR1 |  |
| GPCR | GPCR-formyl peptide | Formyl peptide receptor-like 1 | FPRL1 | FPR2 |  |
| GPCR | GPCR-formyl peptide | Formyl peptide receptor-like 2 | FPRL2 | FPR3 |  |
| GPCR | GPCR-Glycoprotein hormone | Follicle stimulating hormone receptor | FSHR |  |  |
| GPCR | GPCR-fizzled | Frizzled homolog 1 | FZD1 |  |  |
| GPCR | GPCR-fizzled | Frizzled homolog 2 | FZD2 |  |  |
| GPCR | GPCR-frizzled | Frizzled homolog 3 | FZD3 |  |  |
| GPCR | GPCR-frizzled | Frizzled homolog 4 | FZD4 |  |  |
| GPCR | GPCR-fizzled | Frizzled homolog 5 | FZD5 |  |  |
| GPCR | GPCR-fizzled | Frizzled homolog 6 | FZD6 |  |  |
| GPCR | GPCR-fizzled | Frizzled homolog 7 | FZD7 |  |  |
| GPCR | GPCR-frizzled | Frizzled homolog 8 | FZD8 |  |  |
| GPCR | GPCR-fizzled | Frizzled homolog 9 | FZD9 |  |  |
| GPCR | GPCR-frizzled | Frizzled homolog 10 | FZD10 |  |  |
| GPCR | GPCR-GABA | Gamma-aminobutyric acid (GABA) B receptor 1 | GABBR1 |  |  |
| GPCR | GPCR-galanin | Galanin receptor 1 | GALR1 |  |  |
| GPCR | GPCR-galanin | Galanin receptor 2 | GALR2 |  |  |
| GPCR | GPCR-galanin | Galanin receptor 3 | GALR3 |  |  |
| GPCR | GPCR-Glucagon | Growth hormone releasing hormone receptor | GHRHR |  |  |
| GPCR | GPCR-Ghrelin | Growth hormone secretagogue receptor | GHSR |  |  |
| GPCR | GPCR-Glucagon | Gastric inhibitory polypeptide receptor | GIPR |  |  |
| GPCR | GPCR-glucagon | Glucagon-like peptide 1 receptor | GLP1R |  |  |
| GPCR | GPCR-glucagon | Glucagon-like peptide 2 receptor | GLP2R |  |  |
| GPCR | GPCR-GnRH | Gonadotropin-releasing hormone receptor | GNRHR |  |  |
| GPCR | GPCR-Class A Orphans | G protein-coupled receptor 1 | GPR1 |  |  |
| GPCR | Non-signalling 7TM chemokine-binding proteins | G protein-coupled receptor 2 | GPR2 | CCR10 |  |
| GPCR | GPCR-Class A Orphans | G protein-coupled receptor 3 | GPR3 |  |  |
| GPCR | GPCR-Class A Orphans | G protein-coupled receptor 4 | GPR4 |  |  |
| GPCR | GPCR-Class A Orphans | G protein-coupled receptor 6 | GPR6 |  |  |
| GPCR | GPCR-Neuropeptide W | G protein-coupled receptor 8 / Neuropeptides B/W receptor 2 | GPR8 | NPBWR2 |  |
| GPCR | GPCR-Class A Orphans | G protein-coupled receptor 12 | GPR12 |  |  |
| GPCR | GPCR-Class A Orphans | G protein-coupled receptor 15 | GPR15 |  |  |
| GPCR | GPCR-Class A Orphans | G protein-coupled receptor 17 | GPR17 |  |  |
| GPCR | GPCR-Class A Orphans | G protein-coupled receptor 18 | GPR18 |  |  |
| GPCR | GPCR-Class A Orphans | G protein-coupled receptor 19 | GPR19 |  |  |
| GPCR | GPCR-Class A Orphans | G protein-coupled receptor 20 | GPR20 |  |  |
| GPCR | GPCR-Class A Orphans | G protein-coupled receptor 21 | GPR21 |  |  |
| GPCR | GPCR-Class A Orphans | G protein-coupled receptor 22 | GPR22 |  |  |
| GPCR | GPCR-MCH | G protein-coupled receptor 24 | GPR24 | MCHR1 |  |
| GPCR | GPCR-Class A Orphans | G protein-coupled receptor 25 | GPR25 |  |  |
| GPCR | GPCR-Class A Orphans | G protein-coupled receptor 27 | GPR27 |  |  |
| GPCR | GPCR-Class A Orphans | G protein-coupled receptor 31 | GPR31 |  |  |
| GPCR | GPCR-Class A Orphans | G protein-coupled receptor 32 | GPR32 |  |  |
| GPCR | GPCR-Class A Orphans | G protein-coupled receptor 35 | GPR35 |  |  |
| GPCR | GPCR-Class A Orphans | G protein-coupled receptor 37 / Endothelin receptor type B-like | GPR37 |  | ACC/DLPFC/CB |
| GPCR | GPCR-Class A Orphans | G protein-coupled receptor 37-like / Endothelin type B receptor-like protein 2 | GPR37L1 | ET(B)R-LP-2 |  |
| GPCR | GPCR-motilin | G protein-coupled receptor 38 | GPR38 | MLNR |  |
| GPCR | GPCR-Class A Orphans | G protein-coupled receptor 39 | GPR39 |  |  |
| GPCR | GPCR-FFA | G protein-coupled receptor 43 / Free fatty acid receptor 2 | GPR43 | FFAR2 |  |
| GPCR | GPCR-Prostanoid | G protein-coupled receptor 44 | GPR44 | CRTH2 |  |
| GPCR | GPCR-Class A Orphans | G protein-coupled receptor 45 | GPR45 |  |  |
| GPCR | GPCR-Class A Orphans | G protein-coupled receptor 50 | GPR50 |  |  |
| GPCR | GPCR-GABA | G protein-coupled receptor 51 | GPR51 | GABBR2 |  |
| GPCR | GPCR-Class A Orphans | G protein-coupled receptor 52 | GPR52 |  |  |
| GPCR | GPCR-Class B Orphans | G protein-coupled receptor 56 | GPR56 |  | ACC/DLPFC |
| GPCR | GPCR-Class A Orphans | G protein-coupled receptor 58 / Trace amine receptor 2 | GPR58 | TAAR2 |  |
| GPCR | GPCR-Class A Orphans | G protein-coupled receptor 63 | GPR63 |  |  |
| GPCR | GPCR-Class B Orphans | G protein-coupled receptor 64 | GPR64 |  |  |
| GPCR | GPCR-Class A Orphans | G protein-coupled receptor 65 | GPR65 |  |  |
| GPCR | GPCR-Class A Orphans | G protein-coupled receptor 68 | GPR68 |  |  |
| GPCR | GPCR-Class A Orphans | G protein-coupled receptor 75 | GPR75 |  |  |
| GPCR | GPCR-ANAPHYLATOXIN | G protein-coupled receptor 77 / Anaphylatoxin chemotactic receptor C5L2 | GPR77 | C5L2 |  |
| GPCR | GPCR-Class A Orphans | G protein-coupled receptor 85 | GPR85 |  |  |
| GPCR | GPCR-P2Y | G protein-coupled receptor 86 / Purinergic G-protein coupled receptor P2Y 13 | GPR86 | P2RY13 |  |
| GPCR | GPCR-Class A Orphans | G protein-coupled receptor 87 | GPR87 |  |  |
| GPCR | GPCR-Class A Orphans | G-protein coupled receptor 88 | GPR88 |  |  |
| GPCR | GPCR-Class B Orphans | G protein-coupled receptor 97 | GPR97 |  |  |
| GPCR | GPCR-P2Y | G protein-coupled receptor 105 | GPR105 |  |  |
| GPCR | GPCR-Nicotinic acid | G protein-coupled receptor 109B | GPR109B |  |  |
| GPCR | GPCR-Class B Orphans | G protein-coupled receptor 110 | GPR110 |  |  |
| GPCR | GPCR-Class B Orphans | G protein-coupled receptor 116 | GPR116 |  |  |
| GPCR | GPCR-Class B Orphans | G protein-coupled receptor 124 | GPR124 |  |  |
| GPCR | GPCR-Class B Orphans | G protein-coupled receptor 125 | GPR125 |  | ACC |
| GPCR | GPCR-Class B Orphans | G protein-coupled receptor 126 | GPR126 |  |  |
| GPCR | GPCR-Class A Orphans | G protein-coupled receptor 132 | GPR132 |  |  |
| GPCR | GPCR-orphan | G protein-coupled receptor 137 | GPR137 |  |  |
| GPCR | GPCR-orphan | G protein-coupled receptor 137B | GPR137B |  |  |
| GPCR | GPCR-Class B Orphans | G protein-coupled receptor 143 / Ocular albinism type 1 protein | GPR143 | OA1 |  |
| GPCR | GPCR-Class B Orphans | G protein-coupled receptor 144 | GPR144 |  |  |
| GPCR | GPCR-Class A Orphans | G protein-coupled receptor 153 | GPR153 |  |  |
| GPCR | GPCR-Class A Orphans | G protein-coupled receptor 161 | GPR161 |  |  |
| GPCR | GPCR-Class A Orphans | G protein-coupled receptor 162 / Likely ortholog of mouse gene rich cluster, A gene | GPR162 | GRCA |  |
| GPCR | GPCR-Class A Orphans | G protein-coupled receptor 171 / Platelet activating receptor homolog | GPR171 | H963 |  |
| GPCR | GPCR-orphan | G protein-coupled receptor 172B | GPR172B |  |  |
| GPCR | GPCR-orphan | G protein-coupled receptor 176 | GPR176 |  |  |
| GPCR | GPCR-orphan | G protein-coupled receptor 177 | GPR177 |  |  |
| GPCR | GPCR-GPRC5 | G protein-coupled receptor, family C, group 5, member A | GPRC5A | RAI3 / RAIG1 |  |
| GPCR | GPCR-GPRC5 | G protein-coupled receptor, family C, group 5, member B | GPRC5B | RAIG2 | ACC/DLPFC/CB |
| GPCR | GPCR-GPRC5 | G protein-coupled receptor, family C, group 5, member C | GPRC5C | RAIG3 |  |
| GPCR | GPCR-GPRC5 | G protein-coupled receptor, family C, group 5, member D | GPRC5D | RAIG4 |  |
| GPCR | GPCR-GRM | Glutamate receptor, metabotropic 1 | GRM1 |  |  |
| GPCR | GPCR-GRM | Glutamate receptor, metabotropic 2 | GRM2 |  |  |
| GPCR | GPCR-GRM | Glutamate receptor, metabotropic 3 | GRM3 |  | ACC/DLPFC |
| GPCR | GPCR-GRM | Glutamate receptor, metabotropic 4 | GRM4 |  |  |
| GPCR | GPCR-GRM | Glutamate receptor, metabotropic 5 | GRM5 |  |  |
| GPCR | GPCR-GRM | Glutamate receptor, metabotropic 6 | GRM6 |  |  |
| GPCR | GPCR-GRM | Glutamate receptor, metabotropic 7 | GRM7 |  |  |
| GPCR | GPCR-GRM | Glutamate receptor, metabotropic 8 | GRM8 |  |  |
| GPCR | GPCR-bombesin | Gastrin-releasing peptide receptor / Bombesin receptor TYPE 2 | GRPR |  |  |
| GPCR | GPCR-orexin | Hypocretin (Orexin) receptor 1 | HCRTR1 |  |  |
| GPCR | GPCR-orexin | Hypocretin (Orexin) receptor 2 | HCRTR2 |  |  |
| GPCR | GPCR-histamine | Histamine receptor H1 | HRH1 |  |  |
| GPCR | GPCR-histamine | Histamine receptor H2 | HRH2 |  |  |
| GPCR | GPCR-histamine | Histamine receptor H3 | HRH3 |  |  |
| GPCR | GPCR-histamine | Histamine receptor H4 | HRH4 |  |  |
| GPCR | GPCR-HTR | 5-hydroxytryptamine (Serotonin) receptor 1A | HTR1A |  |  |
| GPCR | GPCR-HTR | 5-hydroxytryptamine (Serotonin) receptor 1B | HTR1B |  |  |
| GPCR | GPCR-HTR | 5-hydroxytryptamine (Serotonin) receptor 1D | HTR1D |  |  |
| GPCR | GPCR-HTR | 5-hydroxytryptamine (Serotonin) receptor 1E | HTR1E |  |  |
| GPCR | GPCR-HTR | 5-hydroxytryptamine (Serotonin) receptor 1F | HTR1F |  |  |
| GPCR | GPCR-HTR | 5-hydroxytryptamine (Serotonin) receptor 2A | HTR2A |  |  |
| GPCR | GPCR-HTR | 5-hydroxytryptamine (Serotonin) receptor 2B | HTR2B |  |  |
| GPCR | GPCR-HTR | 5-hydroxytryptamine (Serotonin) receptor 2C | HTR2C |  |  |
| GPCR | GPCR-HTR | 5-hydroxytryptamine (Serotonin) receptor 4 | HTR4 |  |  |
| GPCR | GPCR-HTR | 5-hydroxytryptamine (Serotonin) receptor 5A | HTR5A |  |  |
| GPCR | GPCR-HTR | 5-hydroxytryptamine (Serotonin) receptor 6 | HTR6 |  |  |
| GPCR | GPCR-HTR | 5-hydroxytryptamine (Serotonin) receptor 7 | HTR7 |  |  |
| GPCR | GPCR-chemokine | Interleukin 8 receptor, alpha / Chemokine (C-X-C motif) receptor 1 | IL8RA | CXCR1 |  |
| GPCR | GPCR-chemokine | Interleukin 8 receptor, beta / Chemokine (C-X-C motif) receptor 2 | IL8RB | CXCR2 |  |
| GPCR | GPCR-Glycoprotein hormone | Luteinizing hormone/choriogonadotropin receptor | LHCGR |  |  |
| GPCR | GPCR-Class B Orphans | Latrophilin 1 | LPHN1 |  |  |
| GPCR | GPCR-Class B Orphans | Latrophilin 2 | LPHN2 |  |  |
| GPCR | GPCR-Class B Orphans | Latrophilin 3 | LPHN3 |  |  |
| GPCR | GPCR-Leukotriene | Leukotriene B4 receptor | LTB4R |  |  |
| GPCR | GPCR-Leukotriene | Leukotriene B4 receptor 2 | LTB4R2 | BLTR2 |  |
| GPCR | GPCR-Class A Orphans | MAS1 oncogene | MAS1 |  |  |
| GPCR | GPCR-melanocortin | Melanocortin 1 receptor | MC1R |  |  |
| GPCR | GPCR-melanocortin | Melanocortin 2 receptor | MC2R |  |  |
| GPCR | GPCR-melanocortin | Melanocortin 3 receptor | MC3R |  |  |
| GPCR | GPCR-melanocortin | Melanocortin 4 receptor | MC4R |  |  |
| GPCR | GPCR-melanocortin | Melanocortin 5 receptor | MC5R |  |  |
| GPCR | GPCR-melatonin | Melatonin receptor 1A | MTNR1A |  |  |
| GPCR | GPCR-melatonin | Melatonin receptor 1B | MTNR1B |  |  |
| GPCR | GPCR-bombesin | Neuromedin B receptor / Bombesin receptor type 1 | NMBR |  |  |
| GPCR | GPCR-orphan | Neuropeptide FF 1; RFamide-related peptide receptor | NPFFR1 | GPR147 |  |
| GPCR | GPCR-NPY | Neuropeptide Y receptor Y1 | NPY1R |  | ACC |
| GPCR | GPCR-NPY | Neuropeptide Y receptor Y2 | NPY2R |  |  |
| GPCR | GPCR-NPY | Neuropeptide Y receptor Y5 | NPY5R |  |  |
| GPCR | GPCR-BAR | Nuclear receptor subfamily 1, group H, member 4 / Bile acid receptor | NR1H4 | BAR |  |
| GPCR | GPCR-neurotensin | Neurotensin receptor 1 | NTSR1 |  |  |
| GPCR | GPCR-neurotensin | Neurotensin receptor 2 | NTSR2 |  | ACC |
| GPCR | GPCR-opioid | Opioid receptor, delta 1 | OPRD1 |  |  |
| GPCR | GPCR-opioid | Opioid receptor, kappa 1 | OPRK1 |  |  |
| GPCR | GPCR-opioid | Opiate receptor-like 1 | OPRL1 |  |  |
| GPCR | GPCR-opioid | Opioid receptor, mu 1 | OPRM1 |  |  |
| GPCR | GPCR-oxytocin | Oxytocin receptor | OXTR |  |  |
| GPCR | GPCR-P2Y | Purinergic G-protein coupled receptor P2Y 1 | P2RY1 |  |  |
| GPCR | GPCR-P2Y | Purinergic G-protein coupled receptor P2Y 2 | P2RY2 |  |  |
| GPCR | GPCR-P2Y | Purinergic G-protein coupled receptor P2Y 4 | P2RY4 |  |  |
| GPCR | GPCR-Class A Orphans | Purinergic G-protein coupled receptor P2Y 5 | P2RY5 |  |  |
| GPCR | GPCR-P2Y | Purinergic G-protein coupled receptor P2Y 6 | P2RY6 |  |  |
| GPCR | GPCR-Class A Orphans | Purinergic G-protein coupled receptor P2Y 10 | P2RY10 |  |  |
| GPCR | GPCR-P2Y | Purinergic G-protein coupled receptor P2Y 11 | P2RY11 |  |  |
| GPCR | GPCR-Class A Orphans | Putative neurotransmitter receptor / Trace amine receptor 5 | PNR | TAAR5 |  |
| GPCR | GPCR-NPY | Pancreatic polypeptide receptor 1 / Neuropeptide Y receptor Y4 | PPYR1 | NPY4R |  |
| GPCR | GPCR-PAF | Platelet-activating factor receptor | PTAFR |  |  |
| GPCR | GPCR-Prostanoid | Prostaglandin D2 receptor | PTGDR |  |  |
| GPCR | GPCR-Prostanoid | Prostaglandin E receptor 1 | PTGER1 |  |  |
| GPCR | GPCR-Prostanoid | Prostaglandin E receptor 2 | PTGER2 |  |  |
| GPCR | GPCR-Prostanoid | Prostaglandin E receptor 3 | PTGER3 |  |  |
| GPCR | GPCR-Prostanoid | Prostaglandin E receptor 4 | PTGER4 |  |  |
| GPCR | GPCR-Prostanoid | Prostaglandin F receptor | PTGFR |  |  |
| GPCR | GPCR-Prostanoid | Prostaglandin I2 (Prostacyclin) receptor | PTGIR |  |  |
| GPCR | GPCR-PTH | Parathyroid hormone receptor 1 | PTHR1 |  |  |
| GPCR | GPCR-PTH | Parathyroid hormone receptor 2 | PTHR2 |  |  |
| GPCR | GPCR-GPRC5 | Retinoic acid induced 3 / G protein-coupled receptor, family C, group 5, member A | RAI3 | RAIG1/ GPRC5A |  |
| GPCR | GPCR-Relaxin | Somatostatin- and angiogenin-like peptide receptor / G protein-coupled receptor 135 / Relaxin 3 Receptor 1 | SALPR | GPR135 / RXFP3 / RLN3R1 |  |
| GPCR | GPCR-glucagon | Secretin receptor precursor | SCTR |  |  |
| GPCR | GPCR-Frizzled | Smoothened homolog (Drosophila) | SMO | SMOH |  |
| GPCR | GPCR-Class A Orphans | Super conserved receptor expressed in brain 3 | SREB3 | GPR173 |  |
| GPCR | GPCR-SST | Somatostatin receptor 1 | SSTR1 |  |  |
| GPCR | GPCR-SST | Somatostatin receptor 2 | SSTR2 |  |  |
| GPCR | GPCR-SST | Somatostatin receptor 3 | SSTR3 |  |  |
| GPCR | GPCR-SST | Somatostatin receptor 4 | SSTR4 |  |  |
| GPCR | GPCR-SST | Somatostatin receptor 5 | SSTR5 |  |  |
| GPCR | GPCR-tachykinin | Tachykinin receptor 1 | TACR1 |  |  |
| GPCR | GPCR-tachykinin | Tachykinin receptor 2 | TACR2 |  |  |
| GPCR | GPCR-tachykinin | Tachykinin receptor 3 | TACR3 |  |  |
| GPCR | GPCR-Prostanoid | Thromboxane A2 receptor | TBXA2R |  |  |
| GPCR | GPCR-TRH | Thyrotropin-releasing hormone receptor | TRHR |  |  |
| GPCR | GPCR-Glycoprotein hormone | Thyroid stimulating hormone receptor | TSHR |  |  |
| GPCR | GPCR-VIP and PACAP | Vasoactive intestinal peptide receptor 2 / Hypothetical protein LOC154822 | VIPR2 |  |  |
| GPCR | GPCR-chemokine | Chemokine (C motif) receptor 1 | XCR1 |  |  |
| Neuropeptide ligand |  | Arginine vasopressin / Neurophysin II | AVP |  |  |
| Neuropeptide ligand |  | Calcitonin-related polypeptide, alpha | CALCA |  |  |
| Neuropeptide ligand |  | Calcitonin-related polypeptide, beta | CALCB |  |  |
| Neuropeptide ligand |  | Cholecystokinin | CCK |  |  |
| Neuropeptide ligand |  | Corticotropin releasing hormone | CRH |  |  |
| Neuropeptide ligand |  | Endothelin 2 | EDN2 |  |  |
| Neuropeptide ligand |  | Endothelin 3 | EDN3 |  |  |
| Neuropeptide ligand |  | Galanin | GAL |  |  |
| Neuropeptide ligand |  | Gastrin-releasing peptide | GRP |  |  |
| Neuropeptide ligand |  | Neuromedin B | NMB |  |  |
| Neuropeptide ligand |  | Neuromedin U | NMU |  |  |
| Neuropeptide ligand |  | Neuropeptide FF-amide peptide precursor | NPFF |  |  |
| Neuropeptide ligand |  | Neuropeptide Y | NPY |  | ACC |
| Neuropeptide ligand |  | Neurotensin | NTS |  |  |
| Neuropeptide ligand |  | Oxytocin / neurophysin I | OXT |  |  |
| Neuropeptide ligand |  | Prodynorphin | PDYN |  |  |
| Neuropeptide ligand |  | Proenkephalin | PENK |  | CB |
| Neuropeptide ligand |  | Pro-melanin-concentrating hormone | PMCH |  |  |
| Neuropeptide ligand |  | Prepronociceptin | PNOC |  |  |
| Neuropeptide ligand |  | Proopiomelanocortin | POMC |  |  |
| Neuropeptide ligand |  | Somatostatin | SST |  | ACC/DLPFC |
| Neuropeptide ligand |  | Tachykinin 3 / Neuromedin K / Neurokinin beta) | TAC3 |  |  |
| Neuropeptide ligand |  | Vasoactive intestinal peptide | VIP |  |  |
| G protein | G protein alpha (GNA : G12) | Guanine nucleotide binding protein (G protein) alpha 12 | GNA12 |  |  |
| G protein | G protein alpha (GNA : G12) | Guanine nucleotide binding protein (G protein), alpha 13 | GNA13 |  |  |
| G protein | G protein alpha (GNA) | Guanine nucleotide binding protein (G protein), alpha activating activity polypeptide O | GNAO1 |  |  |
| G protein | G protein alpha (GNA) | Guanine nucleotide binding protein (G protein), alpha transducing activity polypeptide 1 | GNAT1 |  |  |
| G protein | G protein alpha (GNA) | Guanine nucleotide binding protein (G protein), alpha transducing activity polypeptide 2 | GNAT2 |  |  |
| G protein | G protein alpha (GNA) | Guanine nucleotide binding protein, alpha transducing 3 | GNAT3 |  |  |
| G protein | G protein beta (GNB) | Guanine nucleotide binding protein (G protein), beta polypeptide 1 | GNB1 |  |  |
| G protein | G protein beta (GNB) | Guanine nucleotide binding protein (G protein), beta polypeptide 2 | GNB2 |  |  |
| G protein | G protein beta (GNB) | Guanine nucleotide binding protein (G protein), beta polypeptide 3 | GNB3 |  |  |
| G protein | G protein beta (GNB) | Guanine nucleotide binding protein (G protein), beta 5 | GNB5 |  | DLPFC |
| G protein | G protein beta, gamma (GNG) | Guanine nucleotide binding protein (G protein), gamma 10 | GNG10 |  |  |
| G protein | G protein beta, gamma (GNG) | Guanine nucleotide binding protein (G protein), gamma 11 | GNG11 |  |  |
| G protein | G protein beta, gamma (GNG) | Guanine nucleotide binding protein (G protein), gamma 12 | GNG12 |  |  |
| G protein | G protein beta, gamma (GNG) | Guanine nucleotide binding protein (G protein), gamma 13 | GNG13 |  |  |
| G protein | G protein beta, gamma (GNG) | Guanine nucleotide binding protein (G protein), gamma 3 | GNG3 |  |  |
| G protein | G protein beta, gamma (GNG) | Guanine nucleotide binding protein (G protein), gamma 4 | GNG4 |  |  |
| G protein | G protein beta, gamma (GNG) | Guanine nucleotide binding protein (G protein), gamma 5 | GNG5 |  |  |
| G protein | G protein beta, gamma (GNG) | Guanine nucleotide binding protein (G protein), gamma 7 | GNG7 |  |  |
| G protein | G protein beta, gamma (GNG) | Guanine nucleotide binding protein (G protein), gamma transducing activity polypeptide 1 | GNGT1 |  |  |
| Regulator of G proteins |  | Regulator of G-protein signalling 1 | RGS1 |  |  |
| Regulator of G proteins |  | Regulator of G-protein signalling 2 | RGS2 |  |  |
| Regulator of G proteins |  | Regulator of G-protein signalling 3 | RGS3 |  |  |
| Regulator of G proteins |  | Regulator of G-protein signalling 4 | RGS4 |  |  |
| Regulator of G proteins |  | Regulator of G-protein signalling 5 | RGS5 |  |  |
| Regulator of G proteins |  | Regulator of G-protein signalling 6 | RGS6 |  |  |
| Regulator of G proteins |  | Regulator of G-protein signalling 7 | RGS7 |  |  |
| Regulator of G proteins |  | Regulator of G-protein signalling 9 | RGS9 |  |  |
| Regulator of G proteins |  | Regulator of G-protein signalling 10 | RGS10 |  |  |
| Regulator of G proteins |  | Regulator of G-protein signalling 11 | RGS11 |  |  |
| Regulator of G proteins |  | Regulator of G-protein signalling 12 | RGS12 |  |  |
| Regulator of G proteins |  | Regulator of G-protein signalling 13 | RGS13 |  |  |
| Regulator of G proteins |  | Regulator of G-protein signalling 14 | RGS14 |  |  |
| Regulator of G proteins |  | Regulator of G-protein signalling 16 | RGS16 |  |  |
| Regulator of G proteins |  | Regulator of G-protein signalling 17 | RGS17 |  |  |
| Regulator of G proteins |  | Regulator of G-protein signalling 19 | RGS19 |  |  |
| Regulator of G proteins |  | Regulator of G-protein signalling 20 | RGS20 |  | ACC/CB |
| cAMP signaling Core / G protein | G protein alpha (GNA : Gs) | GNAS complex locus | GNAS |  |  |
| cAMP signaling Core (Inhibitory)/ G protein | G protein alpha (GNA : Gi) | Guanine nucleotide binding protein (G protein), alpha inhibiting activity polypeptide 1 | GNAI1 |  | ACC |
| cAMP signaling Core (Inhibitory)/ G protein | G protein alpha (GNA : Gi) | Guanine nucleotide binding protein (G protein), alpha inhibiting activity polypeptide 2 | GNAI2 |  |  |
| cAMP signaling Core (Inhibitory)/ G protein | G protein alpha (GNA : Gi) | Guanine nucleotide binding protein (G protein), alpha inhibiting activity polypeptide 3 | GNAI3 |  |  |
| cAMP signaling Core (Inhibitory)/ G protein | G protein alpha (GNA : Gi) | Guanine nucleotide binding protein (G protein), alpha z polypeptide | GNAZ |  |  |
| cAMP signaling Core | Adenylate cyclase | Adenylate cyclase 1 | ADCY1 |  |  |
| cAMP signaling Core | Adenylate cyclase | Adenylate cyclase 2 | ADCY2 |  |  |
| cAMP signaling Core | Adenylate cyclase | Adenylate cyclase 3 | ADCY3 |  |  |
| cAMP signaling Core | Adenylate cyclase | Adenylate cyclase 6 | ADCY6 |  |  |
| cAMP signaling Core | Adenylate cyclase | Adenylate cyclase 7 | ADCY7 |  |  |
| cAMP signaling Core | Adenylate cyclase | Adenylate cyclase 9 | ADCY9 |  |  |
| cAMP signaling Core (Inhibitory) | Phosphodiesterase | Phosphodiesterase 1A | PDE1A |  | ACC |
| cAMP signaling Core (Inhibitory) | Phosphodiesterase | Phosphodiesterase 1B | PDE1B |  |  |
| cAMP signaling Core (Inhibitory) | Phosphodiesterase | Phosphodiesterase 1C | PDE1C |  |  |
| cAMP signaling Core (Inhibitory) | Phosphodiesterase | Phosphodiesterase 2A | PDE2A |  |  |
| cAMP signaling Core (Inhibitory) | Phosphodiesterase | Phosphodiesterase 3A | PDE3A |  |  |
| cAMP signaling Core (Inhibitory) | Phosphodiesterase | Phosphodiesterase 3B | PDE3B |  |  |
| cAMP signaling Core (Inhibitory) | Phosphodiesterase | Phosphodiesterase 4A | PDE4A |  |  |
| cAMP signaling Core (Inhibitory) | Phosphodiesterase | Phosphodiesterase 4B | PDE4B |  | CB |
| cAMP signaling Core (Inhibitory) | Phosphodiesterase | Phosphodiesterase 4C | PDE4C |  |  |
| cAMP signaling Core (Inhibitory) | Phosphodiesterase | Phosphodiesterase 4D | PDE4D |  |  |
| cAMP signaling Core (Inhibitory) | Phosphodiesterase | Phosphodiesterase 7B | PDE7B |  |  |
| cAMP signaling Core (Inhibitory) | Phosphodiesterase | Phosphodiesterase 8A | PDE8A |  | ACC/DLPFC |
| cAMP signaling Core (Inhibitory) | Phosphodiesterase | Phosphodiesterase 8B | PDE8B |  |  |
| cAMP signaling Core (Inhibitory) | Phosphodiesterase | Phosphodiesterase 9A | PDE9A |  |  |
| cAMP signaling Core (Inhibitory) | Phosphodiesterase | Phosphodiesterase 10A | PDE10A |  |  |
| cAMP signaling Core (Inhibitory) | Phosphodiesterase | Phosphodiesterase 11A | PDE11A |  |  |
| cAMP signaling Core | Protein kinase A (PKA) | Protein kinase, AMP-activated, alpha 1 catalytic subunit | PRKAA1 |  |  |
| cAMP signaling Core | Protein kinase A (PKA) | Protein kinase, AMP-activated, alpha 2 catalytic subunit | PRKAA2 |  |  |
| cAMP signaling Core | Protein kinase A (PKA) | Protein kinase, AMP-activated, beta 2 non-catalytic subunit | PRKAB2 |  |  |
| cAMP signaling Core | Protein kinase A (PKA) | Protein kinase, cAMP-dependent, catalytic, alpha | PRKACA |  |  |
| cAMP signaling Core | Protein kinase A (PKA) | Protein kinase, cAMP-dependent, catalytic, beta | PRKACB |  |  |
| cAMP signaling Core | Protein kinase A (PKA) | Protein kinase, cAMP-dependent, catalytic, gamma | PRKACG |  |  |
| cAMP signaling Core | Protein kinase A (PKA) | Protein kinase, AMP-activated, gamma 1 non-catalytic subunit | PRKAG1 |  |  |
| cAMP signaling Core | Protein kinase A (PKA) | Protein kinase, AMP-activated, gamma 2 non-catalytic subunit | PRKAG2 |  |  |
| cAMP signaling Core | Protein kinase A (PKA) | Protein kinase, cAMP-dependent, regulatory, type I, alpha | PRKAR1A |  |  |
| cAMP signaling Core | Protein kinase A (PKA) | Protein kinase, cAMP-dependent, regulatory, type II, alpha | PRKAR2A |  |  |
| cAMP signaling Core | Protein kinase A (PKA) | Protein kinase, cAMP-dependent, regulatory, type II, beta | PRKAR2B |  |  |
| cAMP signaling Core | PKA anchor | A kinase (PRKA) anchor protein 1 | AKAP1 |  |  |
| cAMP signaling Core | PKA anchor | A kinase (PRKA) anchor protein 5 | AKAP5 |  |  |
| cAMP signaling Core | PKA anchor | A kinase (PRKA) anchor protein 6 | AKAP6 |  |  |
| cAMP signaling Core | PKA anchor | A kinase (PRKA) anchor protein 7 | AKAP7 |  |  |
| cAMP signaling Core | PKA anchor | A kinase (PRKA) anchor protein 8 | AKAP8 |  |  |
| cAMP signaling Core | PKA anchor | A kinase (PRKA) anchor protein 9 | AKAP9 |  |  |
| cAMP signaling Core | PKA anchor | A kinase (PRKA) anchor protein 11 | AKAP11 |  |  |
| cAMP signaling Core | PKA anchor | A kinase (PRKA) anchor protein 12 | AKAP12 |  |  |
| cAMP signaling Core | PKA anchor | A kinase (PRKA) anchor protein 13 | AKAP13 |  |  |
| cAMP signaling Core (Inhibitory) | PKA inhibitor | Protein kinase (cAMP-dependent, catalytic) inhibitor alpha | PKIA |  | ACC |
| cAMP signaling Core (Inhibitory) | PKA inhibitor | Protein kinase (cAMP-dependent, catalytic) inhibitor gamma | PKIG |  |  |
| cAMP signaling Core (Inhibitory) | Protein phosphatase 1 | Protein phosphatase 1, catalytic subunit, alpha isoform | PPP1CA |  | ACC |
| cAMP signaling Core (Inhibitory) | Protein phosphatase 1 | Protein phosphatase 1, catalytic subunit, beta isoform | PPP1CB |  |  |
| cAMP signaling Core (Inhibitory) | Protein phosphatase 1 | Protein phosphatase 1, catalytic subunit, gamma isoform | PPP1CC |  |  |
| cAMP signaling Core (Inhibitory) | Protein phosphatase 1 | Protein phosphatase 1, regulatory (inhibitor) subunit 1A | PPP1R1A |  |  |
| cAMP signaling Core (Inhibitory) | Protein phosphatase 1 | Protein phosphatase 1, regulatory (inhibitor) subunit 2 | PPP1R2 |  |  |
| cAMP signaling Core (Inhibitory) | Protein phosphatase 1 | Protein phosphatase 1, regulatory (inhibitor) subunit 3A | PPP1R3A |  |  |
| cAMP signaling Core (Inhibitory) | Protein phosphatase 1 | Protein phosphatase 1, regulatory (inhibitor) subunit 3C | PPP1R3C |  | ACC/DLPFC |
| cAMP signaling Core (Inhibitory) | Protein phosphatase 1 | Protein phosphatase 1, regulatory subunit 7 | PPP1R7 |  |  |
| cAMP signaling Core (Inhibitory) | Protein phosphatase 1 | Protein phosphatase 1, regulatory (inhibitor) subunit 8 | PPP1R8 |  |  |
| cAMP signaling Core (Inhibitory) | Protein phosphatase 1 | Protein phosphatase 1, regulatory (inhibitor) subunit 9A | PPP1R9A |  |  |
| cAMP signaling Core (Inhibitory) | Protein phosphatase 1 | Protein phosphatase 1, regulatory subunit 10 | PPP1R10 |  |  |
| cAMP signaling Core (Inhibitory) | Protein phosphatase 1 | Protein phosphatase 1, regulatory (inhibitor) subunit 11 | PPP1R11 |  |  |
| cAMP signaling Core (Inhibitory) | Protein phosphatase 1 | Protein phosphatase 1, regulatory (inhibitor) subunit 12A | PPP1R12A |  |  |
| cAMP signaling Core (Inhibitory) | Protein phosphatase 1 | Protein phosphatase 1, regulatory (inhibitor) subunit 12B | PPP1R12B |  |  |
| cAMP signaling Core (Inhibitory) | Protein phosphatase 1 | Protein phosphatase 1, regulatory (inhibitor) subunit 13B | PPP1R13B |  |  |
| cAMP signaling Core (Inhibitory) | Protein phosphatase 1 | Protein phosphatase 1, regulatory (inhibitor) subunit 14D | PPP1R14D |  |  |
| cAMP signaling Core (Inhibitory) | Protein phosphatase 1 | Protein phosphatase 1, regulatory (inhibitor) subunit 16B | PPP1R16B |  |  |
| PI signaling Core / G protein | G protein alpha (GNA : Gq) | Guanine nucleotide binding protein (G protein), q polypeptide | GNAQ |  |  |
| PI signaling Core / G protein | G protein alpha (GNA : Gq) | Guanine nucleotide binding protein (G protein), alpha 11 | GNA11 |  |  |
| PI signaling Core / G protein | G protein alpha (GNA : Gq) | Guanine nucleotide binding protein (G protein), alpha 14 | GNA14 |  |  |
| PI signaling Core / G protein | G protein alpha (GNA : Gq) | Guanine nucleotide binding protein (G protein), alpha 15 | GNA15 |  |  |
| PI signaling Core | Phospholipase C, beta | Phospholipase C, beta 1 | PLCB1 |  |  |
| PI signaling Core | Phospholipase C, beta | Phospholipase C, beta 2 | PLCB2 |  |  |
| PI signaling Core | Phospholipase C, beta | Phospholipase C, beta 3 | PLCB3 |  |  |
| PI signaling Core | Phospholipase C, beta | Phospholipase C, beta 4 | PLCB4 |  |  |
| PI signaling Core | Phosphatidylinositol metabolism (IP3 → I) | Inositol 1,4,5-trisphosphate 3-kinase A | ITPKA |  |  |
| PI signaling Core | Phosphatidylinositol metabolism (IP3 → I) | Inositol 1,4,5-trisphosphate 3-kinase B | ITPKB |  | ACC/DLPFC |
| PI signaling Core | Phosphatidylinositol metabolism (IP3 → I) | Inositol 1,4,5-trisphosphate 3-kinase C | ITPKC |  |  |
| PI signaling Core | Phosphatidylinositol metabolism (IP3 → I) | Inositol polyphosphate-5-phosphatase, 40kDa | INPP5A |  | CB |
| PI signaling Core | Phosphatidylinositol metabolism (IP3 → I) | Inositol polyphosphate-5-phosphatase, 145kDa | INPP5D |  |  |
| PI signaling Core | Phosphatidylinositol metabolism (IP3 → I) | Inositol polyphosphate-5-phosphatase, 72 kDa | INPP5E |  |  |
| PI signaling Core | Phosphatidylinositol metabolism (IP3 → I) | Inositol polyphosphate-5-phosphatase F | INPP5F |  | ACC |
| PI signaling Core | Phosphatidylinositol metabolism (IP3 → I) | Inositol polyphosphate-1-phosphatase | INPP1 |  | ACC |
| PI signaling Core | Phosphatidylinositol metabolism (IP3 → I) | Inositol polyphosphate-4-phosphatase, type I, 107kDa | INPP4A |  |  |
| PI signaling Core | Phosphatidylinositol metabolism (IP3 → I) | Inositol polyphosphate-4-phosphatase, type II, 105kDa | INPP4B |  |  |
| PI signaling Core | Phosphatidylinositol metabolism (IP3 → I) | Inositol(myo)-1(or 4)-monophosphatase 1 | IMPA1 |  |  |
| PI signaling Core | Phosphatidylinositol metabolism (IP3 → I) | Inositol(myo)-1(or 4)-monophosphatase 2 | IMPA2 |  |  |
| PI signaling Core | Phosphatidylinositol metabolism (IP3 → I) | Inositol 1,3,4-triphosphate 5/6 kinase | ITPK1 |  |  |
| PI signaling Core | Phosphatidylinositol metabolism (IP3 → I) | Phosphatase and tensin homolog (mutated in multiple advanced cancers 1) | PTEN |  |  |
| PI signaling Core | Phosphatidylinositol metabolism (DG → I) | Diacylglycerol kinase, alpha | DGKA |  |  |
| PI signaling Core | Phosphatidylinositol metabolism (DG → I) | Diacylglycerol kinase, beta | DGKB |  |  |
| PI signaling Core | Phosphatidylinositol metabolism (DG → I) | Diacylglycerol kinase, delta | DGKD |  |  |
| PI signaling Core | Phosphatidylinositol metabolism (DG → I) | Diacylglycerol kinase, epsilon | DGKE |  |  |
| PI signaling Core | Phosphatidylinositol metabolism (DG → I) | Diacylglycerol kinase, gamma | DGKG |  |  |
| PI signaling Core | Phosphatidylinositol metabolism (DG → I) | Diacylglycerol kinase, iota | DGKI |  |  |
| PI signaling Core | Phosphatidylinositol metabolism (DG → I) | Diacylglycerol kinase, theta | DGKQ |  |  |
| PI signaling Core | Phosphatidylinositol metabolism (DG → I) | Diacylglycerol kinase, zeta | DGKZ |  |  |
| PI signaling Core | Phosphatidylinositol metabolism (DG → I) | CDP-diacylglycerol synthase (phosphatidate cytidylyltransferase) 1 | CDS1 |  | ACC |
| PI signaling Core | Phosphatidylinositol metabolism (DG → I) | CDP-diacylglycerol synthase (phosphatidate cytidylyltransferase) 2 | CDS2 |  |  |
| PI signaling Core | Phosphatidylinositol metabolism (DG → I) | CDP-diacylglycerol--inositol 3-phosphatidyltransferase (phosphatidylinositol synthase) | CDIPT |  |  |
| PI signaling Core | Phosphatidylinositol metabolism (I → PIP2) | Phosphoinositide-3-kinase, class 2, alpha polypeptide | PIK3C2A |  | ACC |
| PI signaling Core | Phosphatidylinositol metabolism (I → PIP2) | Phosphoinositide-3-kinase, class 2, beta polypeptide | PIK3C2B |  | ACC |
| PI signaling Core | Phosphatidylinositol metabolism (I → PIP2) | Phosphoinositide-3-kinase, class 2, gamma polypeptide | PIK3C2G |  |  |
| PI signaling Core | Phosphatidylinositol metabolism (I → PIP2) | Phosphoinositide-3-kinase, class 3 | PIK3C3 |  |  |
| PI signaling Core | Phosphatidylinositol metabolism (I → PIP2) | Phosphoinositide-3-kinase, catalytic, alpha polypeptide | PIK3CA |  |  |
| PI signaling Core | Phosphatidylinositol metabolism (I → PIP2) | Phosphoinositide-3-kinase, catalytic, beta polypeptide | PIK3CB |  |  |
| PI signaling Core | Phosphatidylinositol metabolism (I → PIP2) | Phosphoinositide-3-kinase, catalytic, delta polypeptide | PIK3CD |  |  |
| PI signaling Core | Phosphatidylinositol metabolism (I → PIP2) | Phosphoinositide-3-kinase, catalytic, gamma polypeptide | PIK3CG |  |  |
| PI signaling Core | Phosphatidylinositol metabolism (I → PIP2) | Phosphoinositide-3-kinase, regulatory subunit, polypeptide 1 | PIK3R1 |  | ACC |
| PI signaling Core | Phosphatidylinositol metabolism (I → PIP2) | Phosphoinositide-3-kinase, regulatory subunit, polypeptide 2 | PIK3R2 |  |  |
| PI signaling Core | Phosphatidylinositol metabolism (I → PIP2) | Phosphoinositide-3-kinase, regulatory subunit, polypeptide 3 | PIK3R3 |  |  |
| PI signaling Core | Phosphatidylinositol metabolism (I → PIP2) | Phosphoinositide-3-kinase, regulatory subunit 4 | PIK3R4 |  |  |
| PI signaling Core | Phosphatidylinositol metabolism (I → PIP2) | Phosphatidylinositol-4-Phosphate 5-kinase, type I, beta | PIP5K1B |  |  |
| PI signaling Core | Phosphatidylinositol metabolism (I → PIP2) | Phosphatidylinositol-4-Phosphate 5-kinase, type I, gamma | PIP5K1C |  |  |
| PI signaling Core | Phosphatidylinositol metabolism (I → PIP2) | Phosphatidylinositol-4-Phosphate 5-kinase, type II, gamma | PIP5K2C |  |  |
| PI signaling Core | Phosphatidylinositol metabolism (I → PIP2) | Phosphatidylinositol-3-Phosphate/Phosphatidylinositol 5-kinase, type III | PIP5K3 |  |  |
| PI signaling Core | Protein Kinase C (PKC) | Protein kinase C, alpha | PRKCA |  |  |
| PI signaling Core | Protein Kinase C (PKC) | Protein kinase C, beta 1 | PRKCB1 |  | ACC |
| PI signaling Core | Protein Kinase C (PKC) | Protein kinase C, delta | PRKCD |  |  |
| PI signaling Core | Protein Kinase C (PKC) | Protein kinase C, epsilon | PRKCE |  |  |
| PI signaling Core | Protein Kinase C (PKC) | Protein kinase C, gamma | PRKCG |  |  |
| PI signaling Core | Protein Kinase C (PKC) | Protein kinase C, eta | PRKCH |  |  |
| PI signaling Core | Protein Kinase C (PKC) | Protein kinase C, iota | PRKCI |  | ACC |
| PI signaling Core | Protein Kinase C (PKC) | Protein kinase C, theta | PRKCQ |  |  |
| PI signaling Core | Protein Kinase C (PKC) | Protein kinase C, zeta | PRKCZ |  |  |
| PI signaling Core | Inositol triphosphate (IP3) receptor | Inositol 1,4,5-triphosphate receptor, type 1 | ITPR1 |  | ACC |
| PI signaling Core | Inositol triphosphate (IP3) receptor | Inositol 1,4,5-triphosphate receptor, type 2 | ITPR2 |  |  |
