# Supplementary material for: G protein-linked signaling pathways in bipolar and major depressive disorders
Source: Front Genet. 2013 Dec 23;4:297. doi: 10.3389/fgene.2013.00297 (PMC3870297; doi:10.3389/fgene.2013.00297)
Supplement: Supplementary file 2 [file DataSheet2.DOCX]

The processes of preparation of input files and the Partek analysis are:

Signal intensities were extracted with Robust Multi-array Average (RMA) for each probe set and each subject (Irizarry et al., 2003). Gene-wise Pearson’s correlation coefficients between experimental duplicates were calculated. Genes which show significant correlation coefficient r values (r > 0.423, p < 0.05, n = 22) between two independent sites that ran the U133 Affymetrix microarray chips, were deemed reliably detected and were entered into Partek Genomics (Partek 6.6, St.Charles, MO) resulting in 4,202 genes analyzed.

These reliably measured genes were analyzed in a mixed-model multivariate ANOVA to analyze the main effect of the diagnostic classification (MDD, BD, control) using possible subject effects and experimental sites as factors. Post hoc comparisons (least-squares mean differences) were run to generate p-values for the differences between case and control means. The distributions of p-values were analyzed by the step-up method of false discovery rate (FDR). The FDR was set at the level of accepting 5% false positives among all positives (Benjamini and Hochberg, 1995) for the 4,202 genes analyzed.

The yellow columns indicate the FDR for BD and MDD, and the p-values generated in Partek. The crosstabulations on page 3 indicate the numbers of subjects in each factorial condition.

| BP FDR | MDD FDR | Gene | p-value(Diagnosis) | p-value(Site) | p-value(Subject(Diagnosis)) | p-value(BP vs. Control) | Fold-Change(BP vs. Control) | Estimate (BP vs. Control) | F (BP vs. Control) | T(BP vs. Control) | p-value(MD vs. Control) | Fold-Change(MD vs. Control) | Estimate(MD vs. Control) | F(MD vs. Control) | T(MD vs. Control) | F(Subject(Diagnosis)) | SS(Subject(Diagnosis)) | F(Site) | SS(Site) | F(Diagnosis) | SS(Diagnosis) | SS(Error) | F(Error) |
| --- | --- | --- | --- | --- | --- | --- | --- | --- | --- | --- | --- | --- | --- | --- | --- | --- | --- | --- | --- | --- | --- | --- | --- |
| FDR < 0.05 | FDR < 0.05 | GPR37 | 3.77E-13 | 0.000139 | 2.49E-11 | 9.46E-08 | 1.38298 | 0.467777 | 62.8976 | 7.9308 | 8.39E-09 | -1.4052 | -0.49077 | 84.3783 | -9.18577 | 31.41 | 13.4153 | 21.5855 | 0.485225 | 149.594 | 6.7255 | 0.472062 | 1 |
| FDR < 0.05 | FDR > 0.05 | NPY | 2.07E-06 | 4.11E-09 | 5.73E-05 | 1.48E-06 | 1.33042 | 0.411883 | 43.8575 | 6.6225 | 0.37116 | 1.03633 | 0.051485 | 0.835153 | 0.913867 | 6.2312 | 2.95914 | 91.7079 | 2.29217 | 26.0171 | 1.30056 | 0.52488 | 1 |
| FDR < 0.05 | FDR > 0.05 | PPP1CA | 3.57E-06 | 1.49E-05 | 0.037688 | 3.61E-06 | 1.16846 | 0.224613 | 38.6761 | 6.21901 | 0.627546 | 1.01123 | 0.016109 | 0.242457 | 0.492399 | 2.24363 | 0.359309 | 31.3224 | 0.264008 | 24.1753 | 0.407533 | 0.177003 | 1 |
| FDR < 0.05 | FDR < 0.05 | GPRC5B | 1.63E-12 | 0.039271 | 6.86E-08 | 6.13E-06 | 1.24556 | 0.3168 | 35.805 | 5.98373 | 2.94E-09 | -1.38345 | -0.46828 | 95.3432 | -9.76439 | 13.7118 | 4.71856 | 4.83286 | 0.087532 | 128.777 | 4.66476 | 0.380348 | 1 |
| FDR < 0.05 | FDR > 0.05 | INPP1 | 3.79E-06 | 0.188221 | 0.000756 | 9.4E-06 | 1.24928 | 0.321096 | 33.5929 | 5.79594 | 0.761718 | -1.01074 | -0.01542 | 0.094371 | -0.3072 | 4.38685 | 1.65297 | 1.84987 | 0.036686 | 23.9752 | 0.950935 | 0.416464 | 1 |
| FDR < 0.05 | FDR < 0.05 | SST | 1.21E-07 | 0.000656 | 1.64E-11 | 5.4E-05 | 1.22122 | 0.28832 | 25.4401 | 5.04382 | 0.002288 | -1.13264 | -0.17968 | 12.0422 | -3.47018 | 32.7614 | 13.1426 | 15.97 | 0.337186 | 37.3581 | 1.57754 | 0.443389 | 1 |
| FDR < 0.05 | FDR > 0.05 | GRM3 | 4.6E-06 | 0.004357 | 0.035757 | 0.000113 | 1.34366 | 0.426172 | 22.3969 | 4.73254 | 0.081528 | -1.10899 | -0.14925 | 3.34783 | -1.82971 | 2.26887 | 2.25882 | 10.2059 | 0.534772 | 23.3452 | 2.44651 | 1.10037 | 1 |
| FDR < 0.05 | FDR > 0.05 | EDG2 | 0.000625 | 6.38E-05 | 1.3E-09 | 0.000195 | 1.18211 | 0.241369 | 20.285 | 4.50389 | 0.1806 | 1.0477 | 0.067232 | 1.91814 | 1.38497 | 20.9315 | 7.38033 | 24.7351 | 0.459024 | 10.7016 | 0.397192 | 0.389709 | 1 |
| FDR < 0.05 | FDR > 0.05 | GNAI1 | 0.000149 | 0.000895 | 0.185542 | 0.000289 | 1.40448 | 0.490039 | 18.8311 | 4.33948 | 0.740184 | -1.02411 | -0.03437 | 0.112915 | -0.33603 | 1.49502 | 2.34057 | 14.9456 | 1.23151 | 13.7969 | 2.2737 | 1.73038 | 1 |
| FDR < 0.05 | FDR < 0.05 | PPP1R3C | 1.85E-12 | 4.85E-06 | 9.87E-10 | 0.000544 | -1.15442 | -0.20717 | 16.5993 | -4.07423 | 6.98E-13 | -1.63192 | -0.70657 | 235.315 | -15.34 | 21.5383 | 6.83718 | 37.0629 | 0.61923 | 127.113 | 4.24749 | 0.350858 | 1 |
| FDR < 0.05 | FDR < 0.05 | PDE1A | 0.001993 | 2.83E-05 | 0.000388 | 0.000599 | 1.24131 | 0.31186 | 16.2727 | 4.03395 | 0.01141 | 1.14405 | 0.194155 | 7.68696 | 2.77254 | 4.82341 | 3.53918 | 28.2985 | 1.09285 | 8.48368 | 0.655253 | 0.810987 | 1 |
| FDR < 0.05 | FDR < 0.05 | NPY1R | 0.000909 | 1.15E-05 | 1.51E-05 | 0.000889 | 1.24356 | 0.314474 | 14.9675 | 3.86878 | 0.000855 | 1.21929 | 0.286039 | 15.0919 | 3.88483 | 7.36864 | 5.97721 | 32.5771 | 1.39081 | 9.95666 | 0.85016 | 0.896554 | 1 |
| FDR < 0.05 | FDR > 0.05 | PKIA | 0.004572 | 0.00187 | 0.000116 | 0.001318 | 1.16555 | 0.221007 | 13.7146 | 3.70333 | 0.025332 | 1.09441 | 0.130152 | 5.79679 | 2.40765 | 5.68555 | 2.48594 | 12.6417 | 0.290918 | 7.04043 | 0.324036 | 0.483263 | 1 |
| FDR < 0.05 | FDR < 0.05 | PIK3C2B | 5.03E-06 | 0.009387 | 4.21E-05 | 0.001516 | 1.14765 | 0.198687 | 13.28 | 3.64417 | 0.005757 | -1.11097 | -0.15182 | 9.4495 | -3.074 | 6.48238 | 2.36572 | 8.17633 | 0.157048 | 23.059 | 0.885821 | 0.403361 | 1 |
| FDR < 0.05 | FDR < 0.05 | INPP5F | 0.000216 | 0.210609 | 0.119396 | 0.002445 | 1.16228 | 0.216952 | 11.8472 | 3.44198 | 6.25E-05 | 1.21794 | 0.284447 | 24.8201 | 4.98197 | 1.70139 | 0.829864 | 1.66764 | 0.042811 | 12.9613 | 0.665471 | 0.539099 | 1 |
| FDR < 0.05 | FDR < 0.05 | PDE8A | 2.07E-08 | 0.044827 | 1.55E-08 | 0.004505 | 1.12526 | 0.170252 | 10.1144 | 3.18031 | 3.96E-06 | -1.23079 | -0.29958 | 38.1675 | -6.17799 | 16.1108 | 5.6683 | 4.5525 | 0.084301 | 46.1274 | 1.70833 | 0.388867 | 1 |
| FDR < 0.05 | FDR < 0.05 | RGS20 | 3.41E-08 | 0.32537 | 1.24E-07 | 0.005655 | 1.15621 | 0.209407 | 9.49773 | 3.08184 | 5.85E-06 | -1.29199 | -0.36959 | 36.0583 | -6.00485 | 12.8435 | 7.28002 | 1.01417 | 0.030256 | 43.5007 | 2.59551 | 0.626493 | 1 |
| FDR < 0.05 | FDR < 0.05 | PIK3C2A | 4.5E-06 | 0.000369 | 0.003856 | 0.020892 | 1.22258 | 0.28993 | 6.23734 | 2.49747 | 0.000393 | -1.35916 | -0.44272 | 17.7246 | -4.21006 | 3.41611 | 5.65207 | 17.9513 | 1.56322 | 23.4181 | 4.07853 | 1.8287 | 1 |
| FDR>0.05 | FDR < 0.05 | ITPKB | 0.000303 | 2.09E-06 | 6.8E-08 | 0.035537 | 1.11515 | 0.157241 | 5.04781 | 2.24673 | 0.015198 | -1.12317 | -0.16758 | 6.98739 | -2.64337 | 13.7251 | 8.25335 | 41.7978 | 1.32287 | 12.2148 | 0.773175 | 0.664632 | 1 |
| FDR>0.05 | FDR < 0.05 | GPR125 | 0.035776 | 0.000184 | 0.002807 | 0.088684 | -1.08055 | -0.11177 | 3.18689 | -1.78519 | 0.011594 | -1.11484 | -0.15683 | 7.64735 | -2.76538 | 3.59524 | 1.73014 | 20.5104 | 0.519487 | 3.91923 | 0.198532 | 0.531888 | 1 |
| FDR>0.05 | FDR < 0.05 | ITPR1 | 8.5E-05 | 1.22E-07 | 1.89E-05 | 0.123943 | 1.05462 | 0.076718 | 2.56855 | 1.60267 | 2.66E-05 | 1.17429 | 0.231793 | 28.5763 | 5.34568 | 7.16708 | 2.01623 | 60.9227 | 0.902034 | 15.1369 | 0.448241 | 0.31093 | 1 |
| FDR>0.05 | FDR < 0.05 | GPR56 | 0.000242 | 5.97E-06 | 0.047891 | 0.161404 | 1.07176 | 0.099981 | 2.10699 | 1.45155 | 0.00261 | -1.15911 | -0.21302 | 11.6567 | -3.41419 | 2.1294 | 1.24029 | 35.9512 | 1.10211 | 12.7055 | 0.778992 | 0.643769 | 1 |
| FDR>0.05 | FDR < 0.05 | EDG1 | 1.02E-06 | 1.52E-06 | 6.64E-06 | 0.194276 | -1.04098 | -0.05794 | 1.79803 | -1.34091 | 5.44E-07 | -1.21197 | -0.27735 | 50.2094 | -7.08586 | 8.13932 | 1.86581 | 43.68 | 0.526997 | 28.5831 | 0.689707 | 0.253364 | 1 |
| FDR>0.05 | FDR < 0.05 | NTSR2 | 0.000239 | 0.294523 | 6.69E-07 | 0.741755 | -1.01407 | -0.02016 | 0.111499 | -0.33392 | 0.000195 | -1.18612 | -0.24625 | 20.2789 | -4.50321 | 10.6387 | 4.75972 | 1.15586 | 0.027217 | 12.7353 | 0.599763 | 0.494492 | 1 |
| FDR>0.05 | FDR < 0.05 | EDNRB | 1.04E-08 | 3.31E-06 | 5.04E-06 | 0.765956 | -1.01037 | -0.01489 | 0.090936 | -0.30156 | 1.9E-08 | -1.31161 | -0.39134 | 76.5902 | -8.75158 | 8.41199 | 2.51673 | 39.1536 | 0.616534 | 49.9685 | 1.57366 | 0.330677 | 1 |
| FDR>0.05 | FDR < 0.05 | PRKCB1 | 0.00311 | 0.187059 | 0.001735 | 0.886704 | -1.00527 | -0.00758 | 0.020799 | -0.14422 | 0.00341 | 1.1151 | 0.15717 | 10.8897 | 3.29996 | 3.87547 | 1.31538 | 1.86006 | 0.033228 | 7.69567 | 0.274947 | 0.375139 | 1 |

**Crosstabulations**

| **Factors: 2. Subject; 3. Site; 5. Diagnosis** |
| --- |

| **2. Subject vs. 3. Site** |
| --- |

| **Subject\Site** | **I** | **M** | **Total** |
| --- | --- | --- | --- |
| **1881** | 1 | 1 | 2 |
| **2169** | 1 | 1 | 2 |
| **2208** | 1 | 1 | 2 |
| **2267** | 1 | 1 | 2 |
| **2292** | 1 | 1 | 2 |
| **2311** | 1 | 1 | 2 |
| **2315** | 1 | 1 | 2 |
| **2316** | 1 | 1 | 2 |
| **2466** | 1 | 1 | 2 |
| **2566** | 1 | 1 | 2 |
| **2805** | 1 | 1 | 2 |
| **2861** | 1 | 1 | 2 |
| **2944** | 1 | 1 | 2 |
| **3004** | 1 | 1 | 2 |
| **3018** | 1 | 1 | 2 |
| **3031** | 1 | 1 | 2 |
| **3038** | 1 | 1 | 2 |
| **3064** | 1 | 1 | 2 |
| **3071** | 1 | 1 | 2 |
| **3168** | 1 | 1 | 2 |
| **3196** | 1 | 1 | 2 |
| **3241** | 1 | 1 | 2 |
| **Total** | 22 | 22 | 44 |

| **2. Subject vs. 5. Diagnosis *** |
| --- |

| **Subject\Diagnosis** | **BP** | **Control** | **MD** | **Total** |
| --- | --- | --- | --- | --- |
| **1881** | 2 | 0 | 0 | 2 |
| **2169** | 0 | 2 | 0 | 2 |
| **2208** | 0 | 0 | 2 | 2 |
| **2267** | 0 | 0 | 2 | 2 |
| **2292** | 0 | 2 | 0 | 2 |
| **2311** | 2 | 0 | 0 | 2 |
| **2315** | 0 | 0 | 2 | 2 |
| **2316** | 0 | 2 | 0 | 2 |
| **2466** | 2 | 0 | 0 | 2 |
| **2566** | 2 | 0 | 0 | 2 |
| **2805** | 0 | 2 | 0 | 2 |
| **2861** | 0 | 2 | 0 | 2 |
| **2944** | 0 | 0 | 2 | 2 |
| **3004** | 0 | 0 | 2 | 2 |
| **3018** | 0 | 2 | 0 | 2 |
| **3031** | 0 | 0 | 2 | 2 |
| **3038** | 2 | 0 | 0 | 2 |
| **3064** | 0 | 0 | 2 | 2 |
| **3071** | 0 | 0 | 2 | 2 |
| **3168** | 0 | 0 | 2 | 2 |
| **3196** | 0 | 2 | 0 | 2 |
| **3241** | 2 | 0 | 0 | 2 |
| **Total** | 12 | 14 | 18 | 44 |

| * 2. Subject is nested in 5. Diagnosis. |
| --- |

| **3. Site vs. 5. Diagnosis** |
| --- |

| **Site\Diagnosis** | **BP** | **Control** | **MD** | **Total** |
| --- | --- | --- | --- | --- |
| **I** | 6 | 7 | 9 | 22 |
| **M** | 6 | 7 | 9 | 22 |
| **Total** | 12 | 14 | 18 | 44 |
